# Supplementary material for: Predicting RNA structure and dynamics with deep learning and solution scattering
Source: Biophys J. 2024 Dec 25;124(3):549–64. doi: 10.1016/j.bpj.2024.12.024 (PMC11866959; doi:10.1016/j.bpj.2024.12.024)
Supplement: Document S2. Article plus supporting material [file mmc2.pdf]

Biophysical Journal
Article

# Predicting RNA structure and dynamics with deep learning and solution scattering

Edan Patt,[1] Scott Classen,[2] Michal Hammel,[2,*] and Dina Schneidman-Duhovny[1,*]
[1]School of Computer Science and Engineering, The Hebrew University of Jerusalem, Jerusalem, Israel and [2]Molecular Biophysics and Integrated Bioimaging, Lawrence Berkeley National Laboratory, Berkeley, California

ABSTRACT Advanced deep learning and statistical methods can predict structural models for RNA molecules. However, RNAs are flexible, and it remains difficult to describe their macromolecular conformations in solutions where varying conditions can induce conformational changes. Small-angle x-ray scattering (SAXS) in solution is an efficient technique to validate structural predictions by comparing the experimental SAXS profile with those calculated from predicted structures. There are two main challenges in comparing SAXS profiles to RNA structures: the absence of cations essential for stability and charge neutralization in predicted structures and the inadequacy of a single structure to represent RNA's conformational plasticity. We introduce a solution conformation predictor for RNA (SCOPER) to address these challenges. This pipeline integrates kinematics-based conformational sampling with the innovative deep learning model, IonNet, designed for predicting $Mg^{2+}$ ion binding sites. Validated through benchmarking against 14 experimental data sets, SCOPER significantly improved the quality of SAXS profile fits by including $Mg^{2+}$ ions and sampling of conformational plasticity. We observe that an increased content of monovalent and bivalent ions leads to decreased RNA plasticity. Therefore, carefully adjusting the plasticity and ion density is crucial to avoid overfitting experimental SAXS data. SCOPER is an efficient tool for accurately validating the solution state of RNAs given an initial, sufficiently accurate structure and provides the corrected atomistic model, including ions.

SIGNIFICANCE Understanding the behavior of RNA in solution is critical for deciphering its biological functions, yet predicting its macromolecular conformation remains challenging. While advanced computational methods can predict RNA structures, their accuracy in solution is often limited by the absence of stabilizing ions and the failure to account for RNA's conformational flexibility. This study presents SCOPER, an innovative tool that addresses these challenges by integrating deep-learning-based ion binding site prediction with conformational sampling, offering a more reliable approach to validate and refine RNA structures against experimental SAXS data. We provide our source code and a web server that runs the pipeline.

## INTRODUCTION

In recent years, novel and unexpected roles of noncoding RNAs have been discovered in multiple processes, such as signaling, cancer, development, and stress response (1,2). Structural characterization of their solution conformations is critical to understanding their functional role (3). RNA flexibility challenges traditional structural characterization techniques, such as x-ray crystallography and NMR spectroscopy. While progress has been made with RNA structure determination by cryoelectron microscopy, challenges due to instability, heterogeneity, and small size limit widespread application (4). Consequently, RNA structures comprise only ∼3% of all structures in the Protein Data Bank (PDB) (5). Although novel deep learning methods produce highly accurate protein structures, for RNAs these models are not as accurate and can produce a wide variety of structural models with different base pairings (6).

Small-angle x-ray scattering (SAXS) can rapidly provide in-solution structural information on biological macromolecules, describing the size, shape, and dynamics (7–10). This in-solution structural technique is experiencing a revival primarily due to improvements in data collection technologies and computational algorithms (9). Unlike x-ray crystallography and NMR spectroscopy, SAXS is a fast and reliable technique performed under dilute conditions, thus requiring minimal amounts of RNA samples. The technique has provided reliable data on particles ranging from small RNA

*Submitted June 9, 2024, and accepted for publication December 23, 2024.*
*Correspondence: mhammel@lbl.gov or dina.schneidman@mail.huji.ac.il
Editor: Tamar Schlick.
https://doi.org/10.1016/j.bpj.2024.12.024
© 2024 The Authors. Published by Elsevier Inc. on behalf of Biophysical Society.
This is an open access article under the CC BY license (http://creativecommons.org/licenses/by/4.0/).

duplexes at 20 kDa to the large 70S ribosome at 2700 kDa (10–13). SAXS can be an invaluable tool for the structural biologist, supplementing the traditional high-resolution techniques; yet, the method has limitations for RNA that merit attention.

Directly comparing an RNA structure with a SAXS profile requires an accurate calculator of theoretical SAXS profiles from atomistic models. Current SAXS profile calculators do not accurately match the experimental SAXS RNA data within the noise (14). The difference between the theoretical and experimental SAXS profiles is attributed to the ion-induced changes in the hydration layer (15) and the conformational diversity of RNA (16,17). The results from SAXS studies of counterion interactions with rigid RNA duplexes have greatly improved our understanding of highly charged RNAs folding into compact structures (18). Ions, commonly $Mg^{2+}$, also contribute signal to the SAXS profiles with the x-ray scattering length density of $Mg^{2+}$ almost two times larger than $H_2O$. However, the ions are not predicted by structure modeling algorithms and can be easily missed by experimental structure determination methods. In addition to the localization of ions, we need to be able to predict their solution conformations for functional studies of RNA molecules. Direct comparison between experimental or predicted RNA 3D structures and SAXS profiles measured in solution is challenging. A solution conformation can have similar secondary and tertiary structures as the structural model but different overall conformation due to RNA flexibility. Moreover, for larger flexible RNAs to describe conformational heterogeneity, multistate models (two or more conformations and their weights) can be required (19,20).

Novel works for directly predicting RNA structure from SAXS data have recently emerged such as Ernwin (21) and RNAMasonry (22). These works predict a tertiary structure beginning from a secondary structure and sample a coarse tertiary structure guided by SAXS data. However, these approaches do not account for $Mg^{2+}$ ions or take multiple states into account.

Several methods exist to sample the conformational ensemble from a starting RNA structure, assuming the in-solution conformation only varies slightly. These include molecular dynamics simulations (19), Monte Carlo-based methods, such as SimRNA (23), Normal Modes (15,24,25), and robotics-inspired motion planning approach, KGSRNA (26). However, based on a statistical analysis of ions in the PDB, there is only one predictor of metal ion positions in RNA, MetalionRNA (27). MetalionRNA relies on a distance and angle-dependent anisotropic potential describing interactions between metal ions and RNA atom pairs. The statistical potential was calculated using $\sim$100 structures when it was first published. With the availability of novel geometric deep learning methods for protein structures and a larger data set of RNA structures, there is an opportunity to develop more accurate tools that predict metal ion positions. More recently, works leveraging large amounts of available data have approached this problem using deep learning, such as Zhou and Chen (28), which uses 3D convolutional neural networks to comb the 3D structure of RNA as a 3D image. While this approach seems promising, 3D convolutions are not invariant to the arbitrary orientation of the RNA structure. 3D convolutions are also notoriously slow to compute and require large amounts of data to train while also dealing with many sparse voxels that hinder the training process. The newest version of Deepmind's AlphaFold (AlphaFold3 (6)) released a powerful new model that can generate 3D RNA complexes along with predicted ion binding sites; however, their ion binding site prediction has no reported accuracy measures.

We developed a SAXS-based conformation predictor for RNA (SCOPER), which takes an initial RNA structure and a SAXS profile as input and outputs a single or multistate model with improved fit to the SAXS profile and $Mg^{2+}$ ions added to RNA conformations. We used geometric deep learning to train a model named IonNet for $Mg^{2+}$ ion placement based on the atomic neighborhood. We use SAXS profiles to select subsets of ion positions that best fit experimental SAXS profiles to identify further the most probable ion positions from the predicted ones. To address the conformational flexibility of RNA molecules, we sample multiple conformations using KGSRNA, a motion planning algorithm that preserves the secondary structure of the initial structure, followed by the prediction of $Mg^{2+}$ ion positions for each sampled conformation. Finally, we determine single or multistate models that fit the data within the noise. KGSRNA sampling preserves the secondary structure of the initial RNA structure, enabling reliable sampling of plausible conformational changes driven by RNA flexibility in solution. By preserving RNA's secondary structure, this approach minimizes the risk of overfitting SAXS data by models containing broken basepairs, which may happen in SAXS-guided normal mode sampling (29). We benchmarked SCOPER's capability with 14 experimental SAXS data sets, including experimental data of three RNAs with known crystal structures (P4P6, SAM-riboswitch, and LYS-riboswitch) (30–32).

## MATERIALS AND METHODS

### Summary

The input to our method is a PDB format structure of the RNA and a SAXS profile. The structure can be obtained experimentally or using RNA structure prediction tools, such as DeepRNAFold (33), RNAComposer (34), SimRNA (23), or recently released AlphaFold3 (6). The method proceeds in four main stages (Fig. 1). First, we generate 1000 RNA conformations from the input structure using KGSRNA that perturbs the structure while preserving base pairing (26). The base pairing is assigned by RNAview (35) before the KGSRNA sampling. Second, IonNet predicts potential $Mg^{2+}$ positions for each generated conformation. Third, the input SAXS profile selects a combination of $Mg^{2+}$ ion positions that best fit the data using the goodness of fit parameter $\chi^2$. Finally, if a single conformation with

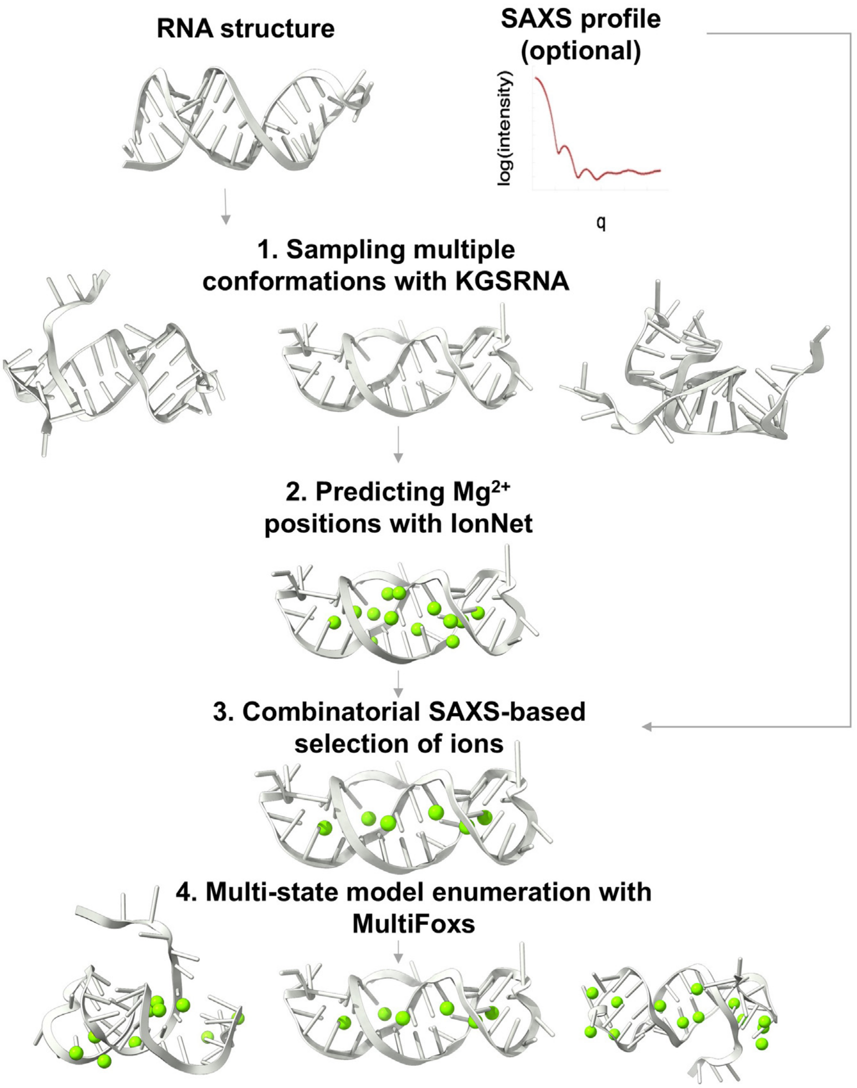


FIGURE 1 Visualization of the SCOPER pipeline

$Mg^{2+}$ ions does not fit the SAXS profile within the noise, we apply MultiFoXS to find multistate models to explore conformational plasticity (36). If a SAXS profile is unavailable, it is still possible to use IonNet as a standalone tool, using the code on GitHub, to obtain the most probable $Mg^{2+}$ ion positions using stages two and three only. The method is available from: https://github.com/dina-lab3d/IonNet. Our pipeline is available for use as a web server: https://bilbomd.bl1231.als.lbl.gov/. Below, we describe stages two and three that we have developed specifically for this pipeline.

## Prediction of ion positions with IonNet

### *IonNet training*

IonNet is a deep learning model that can classify a probe located on an RNA surface as either an $Mg^{2+}$ ion or a water molecule based on the atomic neighborhood of the probe (atoms within an 8 Å radius). To train the model, we relied on ~1000 PDB structures that contain ~41,000 $Mg^{2+}$ ions to serve as positive examples. Water molecules and their atomic neighborhoods were negative examples (supporting material). We describe these neighborhoods as graphs, where nodes correspond to RNA atoms, and edges represent the distances between the nodes. Our model performs its classification using graph neural networks leveraging graph attention (37) and graph convolution (38) layers to extract information from the input graphs (Fig. S1; Table S1). Fourfold cross-validation was performed with IonNet, resulting in an AUROC mean of 0.89 (Fig. 2 *A*). We provide a complete overview of IonNet's training and accuracy metrics in the supporting material (Tables S1 and S3; Figs. S6–S11).

### *IonNet inference*

We generate surface probes to predict ion positions in a given RNA structure (Fig. 2 *B*) using Connolly's surface method (39). A neighborhood of RNA atoms (within 8 Å) is extracted for each such probe. Each neighborhood is passed to IonNet and is classified as either an $Mg^{2+}$ ion or a water molecule neighborhood. When IonNet is used outside of SCOPER as a standalone tool, iterative clustering selects the predicted binding sites with the highest confidence. By the end of this stage, IonNet suggests a number of plausible $Mg^{2+}$ binding site locations. We provide an accuracy metric for this inference process over our test set in the supporting material.

For example, IonNet could identify probes in the vicinity of 11 out of 12 experimentally observed ions for the P4P6 structure (Fig. 2 *C*). Overall, out of ~5500 probes, IonNet identified 84 as $Mg^{2+}$ ions. If we use a strict cutoff (0.9 instead of 0.5) for $Mg^{2+}$ ion prediction, 6 out of the 12 experimentally observed ions are correctly predicted, with 30 predicted $Mg^{2+}$ ion positions (Fig. 2 *D*).

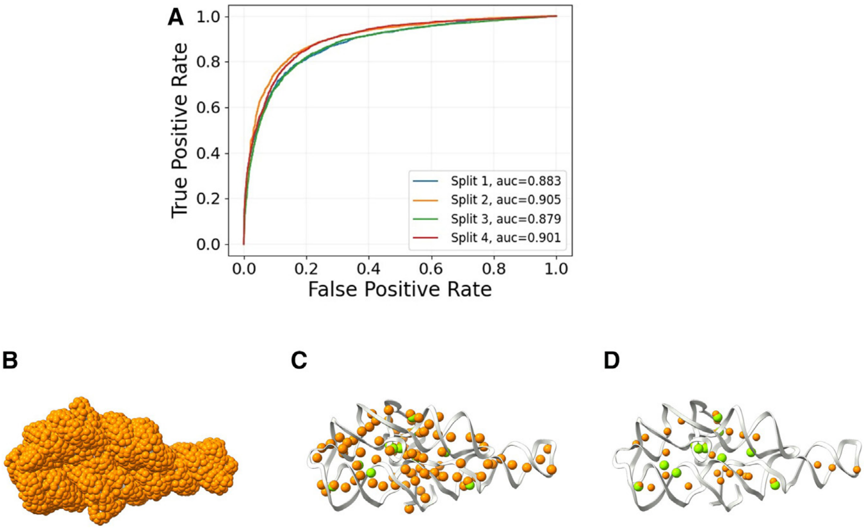


FIGURE 2 IonNet performance. (*A*) Fourfold cross-validation of our best model over the whole test set. (*B–D*) IonNet prediction of $Mg^{2+}$ ions for the P4P6 structure (PDB: 1GID). Ions are predicted from the set of 5500 probes (*orange*) covering the whole structure (*B*) with high-confidence predictions above a threshold of 0.5 with ~80 predictions (*C*) and 0.9 with ~25 predictions (*D*). The experimentally observed $Mg^{2+}$ ions are in green (12 in total).

## Combinatorial SAXS-based selection of ions

### SAXS profile fitting

We computed the profiles using the Debye formula with the FoXS program (14,40). For a given structural model, there are three adjustable parameters: excluded volume ($c_1$), hydration layer density ($c_2$), and the scaling factor ($c$) that are optimized to fit the experimental profile as measured by the $\chi^2$ score:

$$\chi^2 = \frac{1}{S}\sum_{i=1}^{S}\left(\frac{I_{exp}(q_i) - cI(q_i, c_1, c_2)}{\sigma(q_i)}\right)^2$$

where $I_{exp}(q)$ is the experimental profile, which is a function of the momentum transfer $q = 4\pi \sin\theta/\lambda$, where $2\theta$ is the scattering angle, $\lambda$ is the wavelength of the incident x-ray beam, $\sigma(q)$ is the experimental error of the measured profile, and $S$ is the number of points in the profile. $I(q, c_1, c_2)$ is the computed profile, given by the Debye formula (14), with $c_1$ and $c_2$ optimized to minimize the $\chi^2$ score.

## Enumeration of $Mg^{2+}$ subsets

In the pipeline's third stage, the subset of $Mg^{2+}$ ions that minimizes the $\chi^2$ score is selected from a set of predicted $Mg^{2+}$ ion positions by IonNet. These subsets are enumerated using a branch-and-bound algorithm. First, we calculate the $\chi^2$ score for the RNA structure with a single ion. Second, we enumerate all possible subsets with two ions (a branch step) and keep $K$ best scoring ones (a bound step) for the next iteration, when a third ion will be added. We continue enumerating the ion subsets of size $N$ based on size $N-1$ subsets until the score can no longer be improved. To speed up the calculation of the SAXS profile and the corresponding $\chi^2$ score for each subset, we precompute the RNA profile and the profile of each $Mg^{2+}$ ion relative to the RNA. This preprocessing enables a rapid summation of the relevant subprofiles for each subset of $Mg^{2+}$ ions and $\chi^2$ score calculation.

## SAXS data collection

To validate our pipeline, we collect experimental SAXS data from a monomeric RNA state free of contaminants (higher oligomeric states and aggregation). We applied size-exclusion chromatography coupled with SAXS (SEC-SAXS) (Table S2; Fig. S2). All data, except RNA #10 and #14, were collected at SIBYLS beamline 12.3.1 at Advanced Light Source with recently reported developedSEC-SAXS-MALS technique that combines size exclusion chromatography (SEC) with in-line small angle X-ray scattering (SAXS) and multiangle light scattering (MALS) (41). X-ray wavelength was set at $\lambda = 1.127$ Å, and the sample to detector distance was 2100 mm, resulting in scattering vectors, $q$, ranging from 0.01 to 0.4 $Å^{-1}$; 60–95 $\mu$L of annealed RNAs with a concentration between 1 and 3 mg/mL was prepared in the SEC running buffer (Table S2). The Shodex KW802.5 column was equilibrated with a running buffer with a flow rate of 0.65 mL/min. Each sample was injected in an SEC column, and 2-s x-ray exposures were recorded continuously for 24 min. RNA #10 and #14 were previously collected at the SIBYLS beamline and reported (13,32). Program RAW (42) was used for further SEC-SAXS processing, including buffer subtractions and merging SAXS frames across the elution peak (Fig. S2). For RNA #8, #9, and #11, we applied the EFA approach (43) to ensure that the SAXS signal is derived from monomeric and well-folded RNA. The final merged SAXS curves were further used for Guinier analysis using the program RAW (42) and computing P(r) functions by the program GNOM (44). The $MW_{SAXS}$ was calculated using volume of correlation (45) and compared with the molecular weight estimated by SEC-MALS (Table S2). The SEC-SAXS were deposited to the SIMPLE SCATTERING database https://simplescattering.com/ (deposition IDs are listed in Table S2).

# RESULTS

## Impact of ionic strength and RNA plasticity on SAXS fitting

Fast calculation of SAXS profiles from protein structures usually involves modeling the hydration shell with implicit solvent models (14,46–49). In these models, the density of the hydration layer can be adjusted to optimize the fit to the experimental SAXS profile (14,46). The changes in the protein's hydration layer, typically influenced by the protein surface net charge, result from the varying ionic strength of the buffer used in SAXS experiments (14). However, in the nucleic acid world, higher ionic strength increases the rigidity of nucleic acid structures (16) and can alter the overall arrangement of RNA segments (50,51), whereas the hydration layer is poorly understood. The ionic strength-dependent plasticity of RNA poses a greater

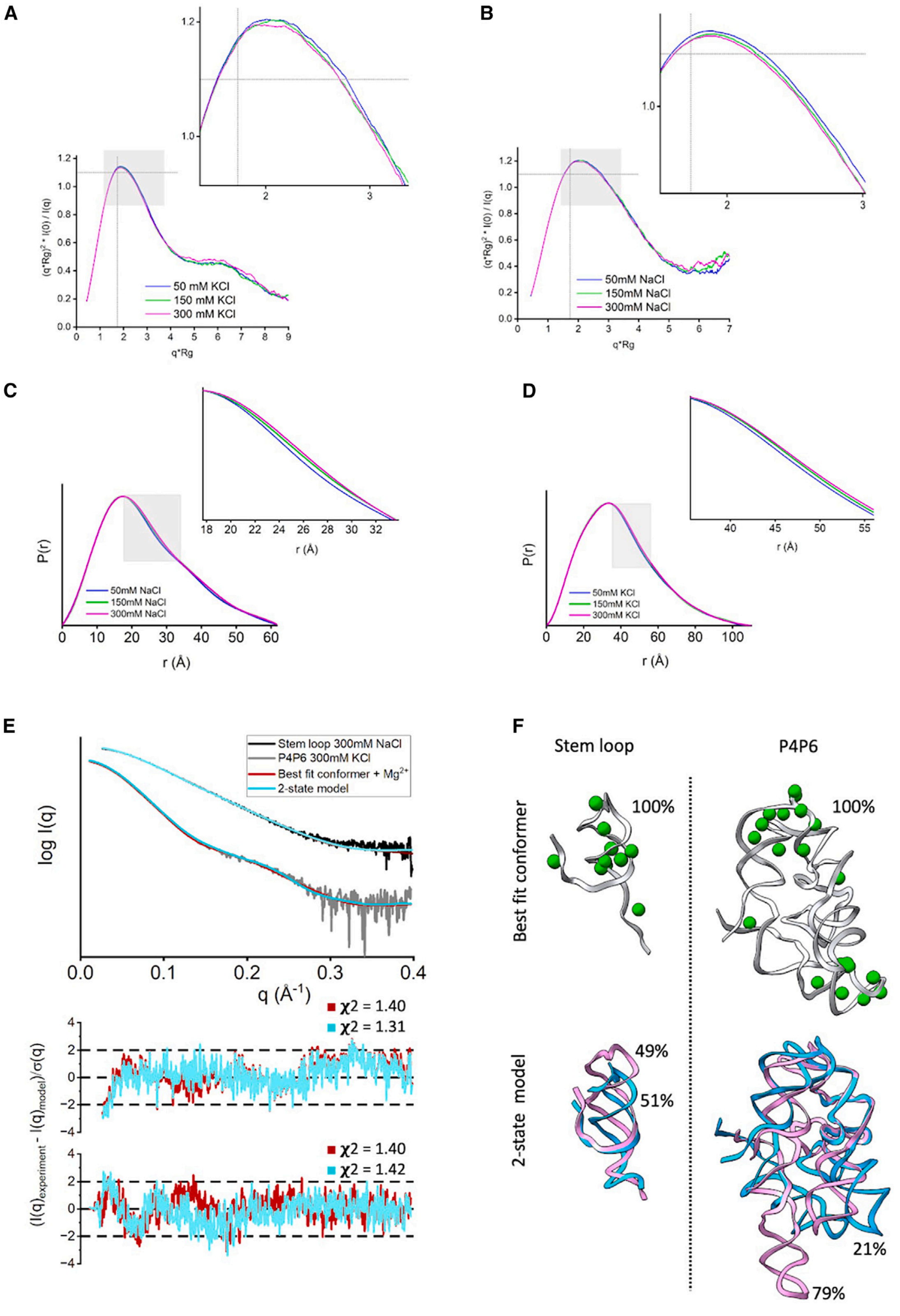


FIGURE 3 Impact of ionic strength on the hydration layer, $Mg^{2+}$ placement, and RNA conformation. (*A* and *B*) Normalized Kratky plot for experimental SAXS curves of two RNAs (#3 RNA stem-loop, #13 P4P6) measured at three different salt concentrations, 50 mM (*blue*), 150 mM (*green*), and 300 mM (*pink*). The SAXS signal was smoothed using adjacent averaging to visualize differences between the curves better. The dashed gray lines on the dimensionless (Rg) plot are guidelines for a globular protein. For a globular protein, the peak position should be at $qRg = 3 \approx 1.73$, while the peak height should be

*(legend continued on next page)*

**TABLE 1 Concentration results**

| Structure/salt concentration | Rg (Å) | Without $Mg^{2+}$ | | | | With $Mg^{2+}$ | | | With $Mg^{2+}$ $c_1 = 1.0/c_2 = 1.0$ | | |
|---|---|---|---|---|---|---|---|---|---|---|---|
| | | Best single conformer | | Two-state model | | Best single conformer | | | Best single conformer | | Multistate model |
| | | $\chi^2$ | $c_2$ | $\chi^2$ | $c_2$ | $\chi^2$ | $c_2$ | No. of ions | $\chi^2$ | No. of ions | $\chi^2$ |
| P4P6-50 | 29.57 ± 0.09 | 1.90 | 2.50 | 1.63 | 2.0 | 1.49 | 2.00 | 14 | 2.45 | 20 | 1.42 with $Mg^{2+}$<br>1.52 without $Mg^{2+}$ |
| P4P6-150 | 29.56 ± 0.11 | 1.96 | 0.56 | 1.39 | 2.0 | 1.63 | 0.85 | 16 | 1.74 | 17 | 1.39 with $Mg^{2+}$<br>1.84 without $Mg^{2+}$ |
| P4P6-300 | 29.61 ± 0.11 | 1.78 | 0.45 | 1.31 | 2.0 | 1.40 | 0.84 | 18 | 1.48 | 20 | 1.17 with $Mg^{2+}$<br>1.21 without $Mg^{2+}$ |
| Stem-loop-50 | 18.15 ± 0.11 | 1.90 | −0.14 | 1.50 | −0.5 | 1.67 | −0.46 | 11 | 1.99 | 9 | 1.82 with $Mg^{2+}$<br>1.45 without $Mg^{2+}$ |
| Stem-loop-150 | 18.15 ± 0.08 | 1.98 | −0.08 | 1.51 | −0.5 | 1.64 | −0.50 | 11 | 1.96 | 9 | 1.72 with $Mg^{2+}$<br>1.37 without $Mg^{2+}$ |
| Stem-loop-300 | 18.14 ± 0.05 | 1.65 | 0.48 | 1.42 | −0.5 | 1.40 | 0.55 | 9 | 1.52 | 10 | 1.22 with $Mg^{2+}$<br>1.08 without $Mg^{2+}$ |

difficulty in quantifying changes in the RNA hydration layer using SAXS. Nevertheless, here, we look at the impact of salt concentration on the hydration layer model in the FoXS SAXS calculator (14) and RNA plasticity.

We measured SAXS profiles for a small RNA stem-loop (#3 in the benchmark) and P4P6 RNA (#13 in the benchmark) at different ionic strengths while keeping the $Mg^{2+}$ concentration constant at 5 mM. Although the radius of gyration (Rg) values obtained by the Guinier plot (Fig. 3, *A* and *B*) are identical within the error across the ionic strengths (Table 1), we observed variations in the SAXS profile shown by the normalized Kratky plot (Fig. 3, *A* and *B*). The narrowing of the peak at higher ionic strength (Fig. 3, *A* and *B*) could be further visualized by changes in the P(r) function (Fig. 3, *C* and *D*). Low concentration of RNAs (stem-loop ~0.06 mM, P4P6 ~0.01 mM) at the elution peak, presence of 5 mM $Mg^{2+}$, and identical Rg values across measured salt concentrations (Table 1) shows that SAXS changes are not derived from altered interparticular RNA interactions (52). Multiple factors may explain the observed changes: 1) changes in RNA rigidity as previously observed for DNA (53–55) and RNA rearrangement (50,51), or 2) changes in the hydration layer influenced by the altered RNA surface net charge, or all of the above. Nevertheless, because distinguishing RNA plasticity/rigidity from the altering hydration layer is difficult, we fit the SAXS profiles with the single or ensemble model of RNA, with/without adjustment of the $c_1/c_2$ parameters or with/without placement of $Mg^{2+}$. The quality of fit for all the fittings is listed in Table 1. The single conformer with adjusted $c_1/c_2$ parameters did not match the SAXS profiles within the noise (Table 1), indicating that adding $Mg^{2+}$ ions or a multistate model (36) is necessary to match the data. However, the fit for a model with placed $Mg^{2+}$ or a two-state model is similar (Table 1; Fig. 3, *E* and *F*) and shows that the RNA plasticity or placement of $Mg^{2+}$ is challenging to distinguish. The single conformer with placed $Mg^{2+}$ ions and default $c_1/c_2$ parameters slightly worsened the fit. In contrast, multistate models with placed $Mg^{2+}$ ions and default c1/c2 parameters significantly improved the fit for P4P6 but not stem-loop (Table 1). This further indicates that fitting SAXS simultaneously with RNA plasticity, adjustment of the hydration layer and excluded volume, and placing $Mg^{2+}$ may lead to data overfitting. To avoid SAXS data overfitting, we set the c1 and c*2* parameters to the default value of 1.0 in our further pipeline testing.

## SAXS profile fitting

Our data set consisted of 14 RNA samples with experimental SAXS profiles. The starting structures were obtained from DeepFoldRNA (#1–4, #6–8) (33), RNAComposer (#5, #9, #11), (34), and x-ray crystallography structures (#10 PDB: 2GIS, #13, PDB: 1GID, #14 PDB: 3D0U) (31,32,56). We used different programs to predict our initial structures mainly due to time constraints, where RNAComposer produced initial fitting structures faster than DeepFoldRNA. DeepFoldRNA was used when the

$3/e \approx 1.1$. (*B* and *C*) P(r) functions of two RNAs (#3 RNA stem-loop, #13 P4P6) calculated from SAXS curves (see Fig. S4) measured at three different salt concentrations, 50 mM (*blue*), 150 mM (*green*), and 300 mM (*pink*). (*E*) Experimental SAXS curves collected at 300 mM salt concentration for RNA #3 (stem-loop, *black*) and #13 (P4P6, *gray*). SAXS fit of the best-fit structure with $Mg^{2+}$ (*red*) and two-state model without $Mg^{2+}$ (*cyan*) reveal similar goodness of fit. The $c_1$ and $c_2$ parameters were adjusted in both fitting approaches (see Table 1). Bottom panel: SAXS fit residual with its $\chi^2$ values. (*F*) Best-fit conformer with $Mg^{2+}$ and two-state model shown for #3 RNA stem-loop, #13 P4P6 with corresponding weights. The $\chi^2$ and $c_2$ values for all salt concentrations are in Table 1.

**TABLE 2 $\chi^2$ values for our fitting experiments**

| RNA | Starting structure | | Best scoring structure | | MultiFoXS | | |
|---|---|---|---|---|---|---|---|
| | Without $Mg^{2+}$ | With $Mg^{2+}$ | Without $Mg^{2+}$ | With $Mg^{2+}$ | Without $Mg^{2+}$ | With $Mg^{2+}$ | No. of states |
| 1 | 9.29 | 9.29 | 1.55 | 1.07 | 1.35 | 1.06 | 3 |
| 2 | 42.67 | 42.67 | 5.12 | 2.92 | 4.64 | 2.92 | 3 |
| 3 | 18.95 | 10.52 | 1.76 | 1.52 | 1.08 | 1.22 | 6 |
| 4 | 8.70 | 8.70 | 3.04 | 2.47 | 2.94 | 2.41 | 3 |
| 5 | 14.91 | 9.90 | 5.95 | 3.86 | 4.27 | 3.35 | 3 |
| 6 | 2.18 | 1.97 | 2.02 | 1.94 | 1.87 | 1.89 | 3 |
| 7 | 1.92 | 1.55 | 1.03 | 1.00 | 0.96 | 0.95 | 6 |
| 8 | 4.65 | 3.35 | 3.15 | 2.43 | 2.71 | 2.37 | 4 |
| 9 | 8.30 | 8.20 | 2.93 | 2.44 | 2.93 | 2.44 | 2 |
| 10 | 6.51 | 3.33 | 5.60 | 1.77 | 4.00 | 1.77 | 2 |
| 11 | 8.48 | 8.07 | 1.86 | 1.45 | 1.37 | 1.31 | 4 |
| 12 | 5.26 | 3.84 | 3.34 | 2.66 | 3.34 | 2.66 | 2 |
| 13 | 26.49 | 23.24 | 1.86 | 1.48 | 1.21 | 1.17 | 4 |
| 14 | 6.87 | 6.87 | 2.62 | 1.62 | 0.69 | 0.69 | 4 |

RNAComposer model fitted the SAXS data poorly. In addition, we apply the SCOPER pipeline with AlphaFold3 for large RNAs (#9, #11) to show how our pipeline can validate or reject models with different secondary structures (see below). Deep learning prediction models such as DeepFoldRNA or AlphaFold3 offer multiple conformations when predicting 3D structures. We always selected the conformation with the best initial SAXS fit. We found that predictions with an inadequate initial structure were usually unsuitable for our pipeline as KGSRNA explores the conformational space of RNA by preserving its secondary structure. Our pipeline is expected to benefit from future improvements in RNA structure prediction that will provide more accurate starting structures (57). The fit of most starting structures to their corresponding SAXS profile was relatively poor (Table 2; Fig. 4). We predicted $Mg^{2+}$ ion positions using IonNet. Adding $Mg^{2+}$ ions to the starting structure only marginally improved the fit (Table 2; Figs. 4 and 5). However, in the case of the accurately determined starting structure of the SAM-riboswitch (#10) obtained from x-ray crystallography, the addition of $Mg^{2+}$ ions resulted in significant improvement (Table 2; Fig. 4). The SAM-riboswitch is a well-folded globular RNA where the addition of a relatively large number of placed $Mg^{2+}$ ions contributes to the improvement of the SAXS profile fit. Significant improvement in SAXS fit could also be observed for some small RNAs (#3, #5) with more straightforward folds and expected limited plasticity. Thus, the starting predicted structure represents the solution state, and placing $Mg^{2+}$ ions improves the SAXS fit. However, selecting a more accurate conformation is critical to matching the experimental SAXS profiles except for the RNA-riboswitch (#10).

In all of our benchmark cases, the single conformation with the best fit (lowest $\chi^2$) out of the 1000 sampled by KGSRNA could fit the data significantly better than the initial structure (Table 2; Figs. 4 and 5). Adding $Mg^{2+}$ ions to these conformations further improved the fit (Table 2; Figs. 4 and 5). Additional improvement in the fit to SAXS profiles could be achieved using multistate models that mimic RNA plasticity (Table 2). Notably, the multisegment RNAs where the more significant movement of segments could be expected (#11, #13, and #14) (50) showed significant fit improvement with a multistate model. For example, the SAXS profile of the P4P6 had the best fit with four conformations (Fig. S3) with a $\chi^2 = 1.17$ vs. 1.48 for a single conformation. However, the different placements of $Mg^{2+}$ in each conformer (Fig. 3) suggest that placing $Mg^{2+}$ ions in combination with multistate modeling can lead to the overfitting of experimental SAXS data. On the other hand, it may also indicate that the movement of individual P4P6 segments in solution can result in more loosely defined coordinates for $Mg^{2+}$ ions. Generally, validating through SAXS whether the $Mg^{2+}$ ions observed in the P4P6 crystal structure remain ordered in the dynamic solution state is challenging. Smaller RNAs with single-stranded regions (#3) also show improved fit with a multistate model. The multistate model of the #3 RNA mimics the plasticity of the unpaired 5′ end, resulting in a $\chi^2$ value of 1.22 compared with 1.52 for a single conformation (Table 2).

These results indicate that our pipeline can predict $Mg^{2+}$ binding sites that significantly reduce the $\chi^2$ score of the RNA structure (Figs. 4 and 5; Table 2). Although the improvements to the fit by adding $Mg^{2+}$ ions were relatively marginal compared with finding a suitable conformation, these additions are not insignificant (Table 2; Figs. 4 and 5).

We conducted our profile fit calculations over all 1000 conformations generated by KGSRNA. We found that running IonNet on all 1000 conformations can be redundant if a single conformation fits the data within the noise. The best fitting conformation without $Mg^{2+}$ ions tends to be one of the best fitting conformations with added ions (Fig. S5). If a user lacks parallel computing power, a safe assumption is that our pipeline can be run only on the best scoring structures sampled by KGSRNA with little cost to the optimality of the output. However, if multistate modeling is needed to fit the data within the noise, one needs to run IonNet for all the conformations.

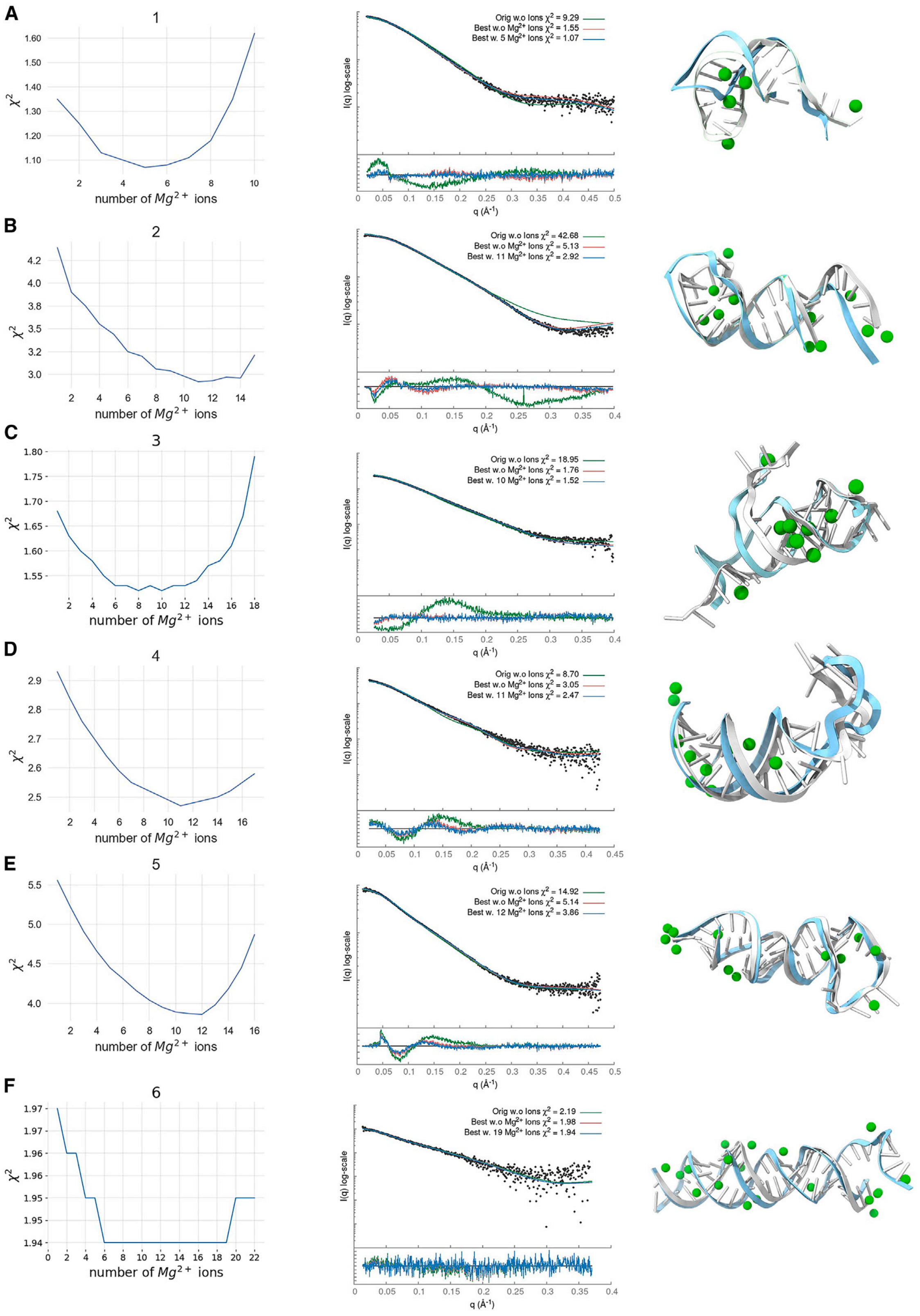


*(figure continued on next page)*

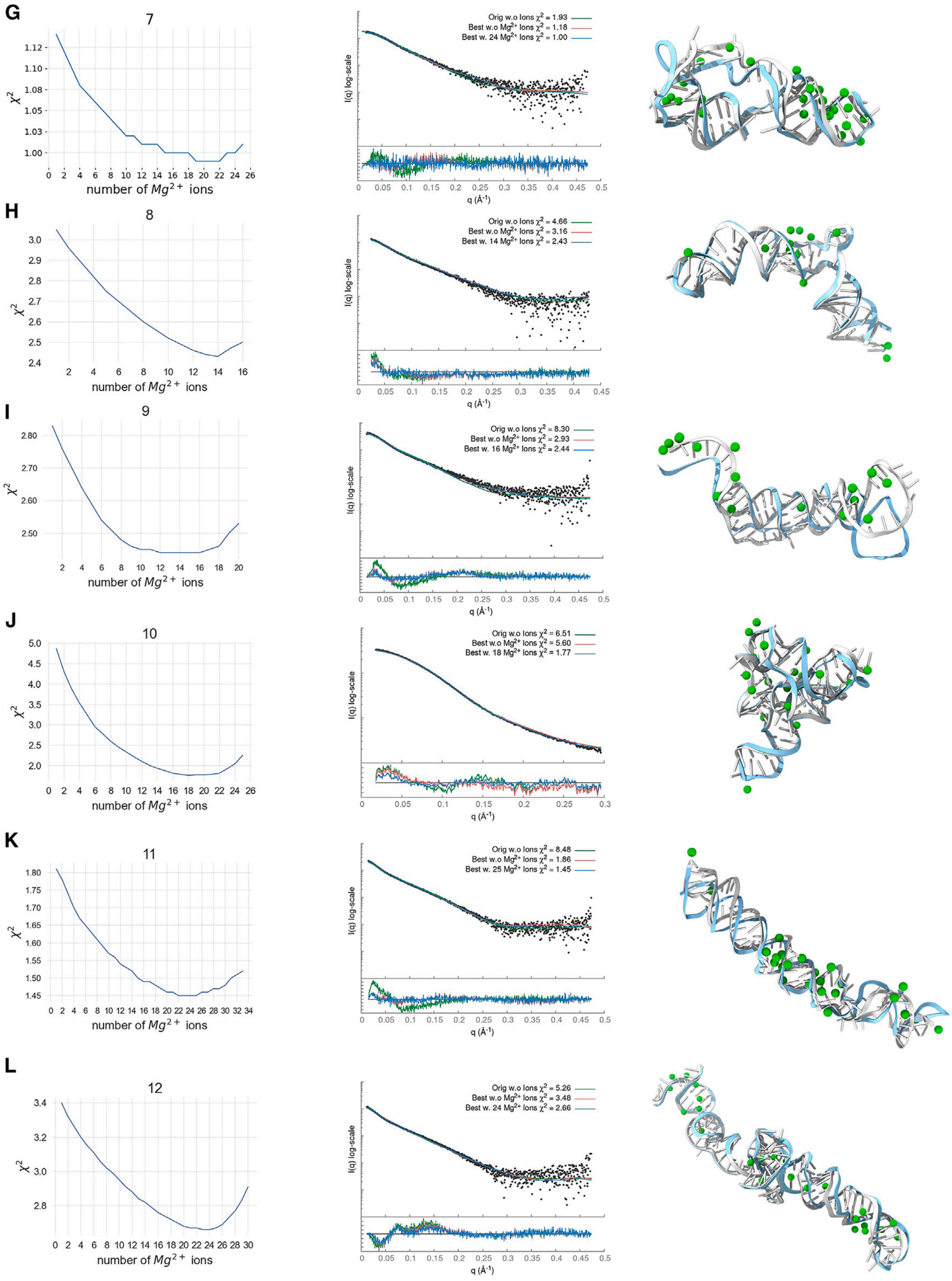


(figure continued on next page)

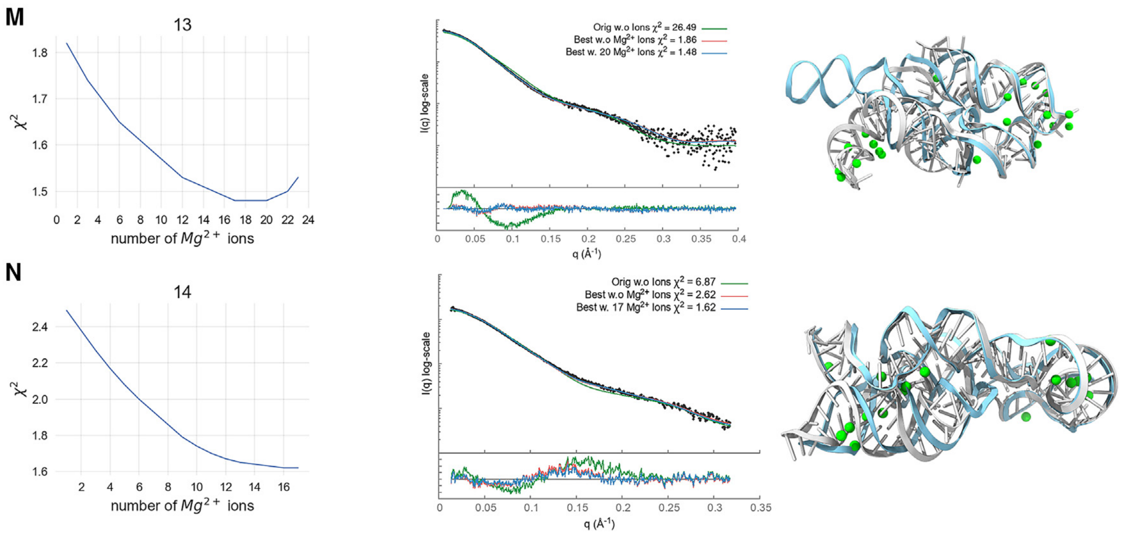


FIGURE 4 SCOPER results for 14 benchmark cases (labeled *A–N*). The decrease in $\chi^2$ per $Mg^{2+}$ ion added (*left column plots*), SAXS profile fit of original starting structure (*green*), best structure without $Mg^{2+}$ ions (*red*), and best structure with added $Mg^{2+}$ ions (*blue*) versus experimental profile (*black dots*) (*middle column plots*), initial RNA structure (*cyan*) and best fitting RNA structure (*white*) with predicted $Mg^{2+}$ ions (*green*) (*right column*).

## Using SCOPER for validation of structural models

We observed that the SCOPER can validate structures with adequate secondary and tertiary initial structures for the SAXS profile. We find that only adding $Mg^{2+}$ ions predicted by IonNet is insufficient to fit the data (Table 2; Fig. S5) as most structures' fit to the profile is usually only marginally changed when $Mg^{2+}$ ions are added. IonNet selects binding sites with high accuracy, and SCOPER selects ions that reduce the SAXS score with a clustering algorithm; both help to reduce overfitting the SAXS profile by only adding ions, although these do not eliminate the possibility that some ions are chosen specifically because they help overfit the SAXS profile.

During experimentation, we also noticed that SCOPER worked best when the initial structure fit relatively well. Because KGSRNA preserves the initial structure's secondary structure, SCOPER is unlikely to overfit a structure with the wrong secondary structure convincingly. While KGSRNA does lend itself to exploring a large conformation space (Fig. S12), we found 1000 iterations to be sufficient for our needs; however, this number can be increased if needed.

We ran SCOPER with two initial models derived from two different prediction programs for three RNAs to demonstrate the role of SCOPER in validating structures rather than using it as a structure modeling tool. We chose two large RNAs (#9, #11) with the unknown experimentally defined structure and possible variations in tertiary structure prediction by AlphaFold3 (6) versus DeepFoldRNA or RNAcomposer. The SCOPER pipeline shows a significantly better fit for the refined DeepFoldRNA models (#9 $\chi^2 = 3.65$ vs. 2.44, #11 $\chi^2 = 5.84$ vs. 1.45) (Fig. 6, *A* and *B*). In case #9, the unfolded RNA 3′-end was the most significant difference contributing to the good fit derived from the DeepFoldRNA model. In case #11, the absence of unpaired stem-loop segments in the AlphaFold3 model leads to a noticeably poorer fit.

When multiple conformations exist in a solution with variations in secondary structure between them, SCOPER can be run with different initial structures. The resulting multistate model can then be determined by combining SCOPER's outputs for all initial structures using MultiFoXS (36). Therefore, we strongly recommend using SCOPER to validate or reject structural models based on a SAXS profile rather than relying on it solely as a predictive tool. This is because SCOPER's effectiveness is greatly dependent on the accuracy of the initial structure, and its limitations must be carefully taken into account.

## Correlation between RNA size and a number of potential $Mg^{2+}$ binding sites

We found a positive correlation (Pearson coefficient = 0.67) between the number of atoms in an RNA structure and the amount of predicted $Mg^{2+}$ ions for the structure (Fig. 7). This is an expected outcome as the more surface area the structure has, it would seem likely that there would be more $Mg^{2+}$ binding sites to stabilize the structure.

## SCOPER web app

We implemented a straightforward and easy-to-use web-based application to make the SCOPER pipeline available to the scientific community, particularly those without the knowledge or resources to install, configure, and run the

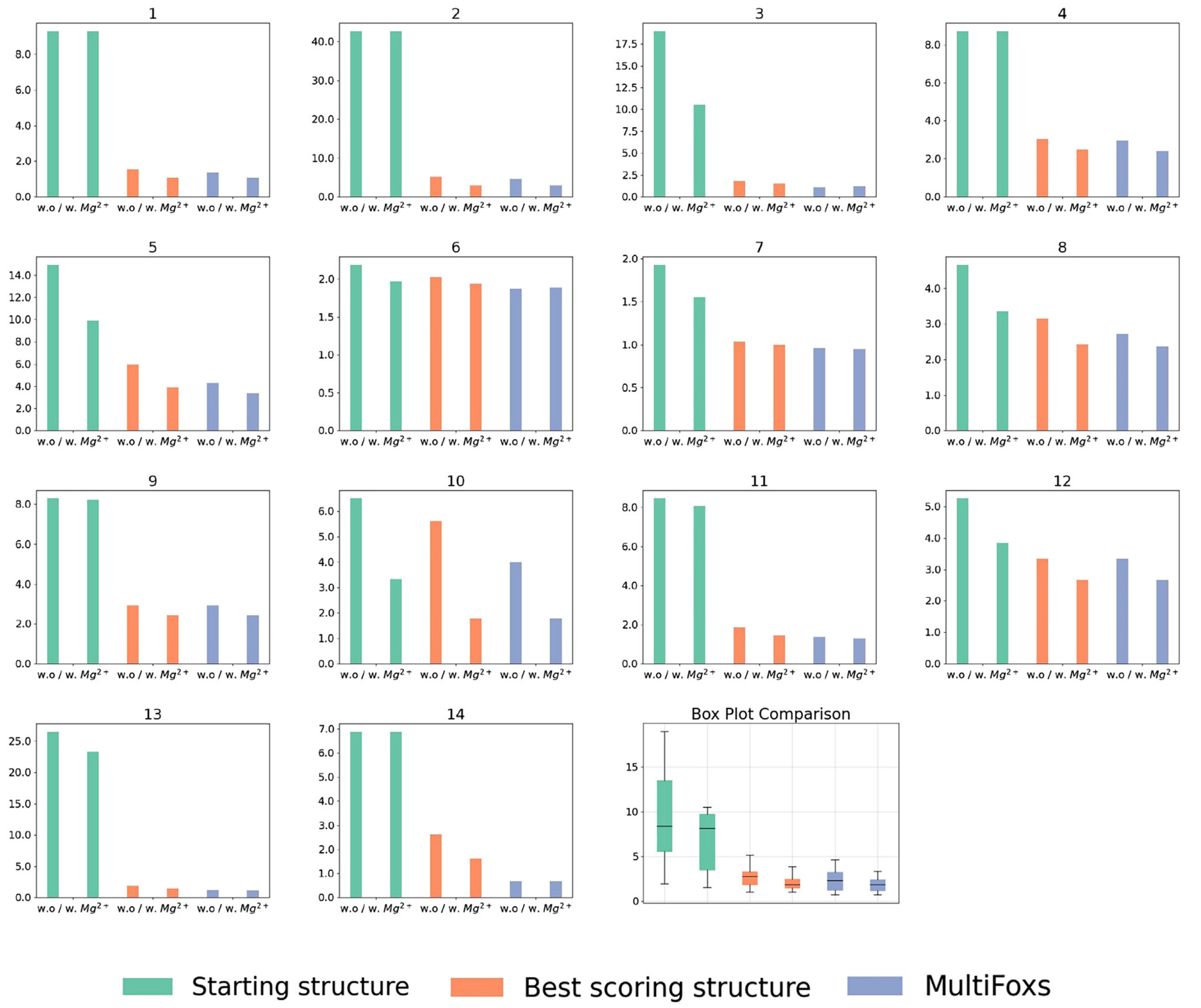


FIGURE 5 Bar plots of $\chi^2$ scores for the 14 benchmark cases: starting structures (*green*), best scoring conformations (*orange*), and multistate models (*purple*), all with and without the addition of $Mg^{2+}$ ions. A summary of the scores for all benchmark cases is provided in the last boxplot.

Python code directly from the GitHub repository. The SCOPER pipeline has been added as an option within our existing BILBOMD job runner framework (58), available at https://bilbomd.bl1231.als.lbl.gov/. Users are required to create an account before submitting jobs. The required inputs are the initial RNA structure in PDB format and the experimental SAXS curve containing three columns ($q$ in $\text{Å}^{-1}$ unit, intensities, and experimental error.). The SCOPER web server implements the following pipeline steps (Fig. 1): 1) conformational sampling by KGSRNA, 2) selection of the best conformer using FoXS, 3) prediction of possible locations of $Mg^{2+}$ ions using IonNet, and 4) selection of the best $Mg^{2+}$ ion placements by fitting the SAXS data using MultiFoXS. Users are notified by email when their jobs are complete. Results are then available to view directly within the web app (Fig. 8). They are also available to download as a compressed file containing the original uploaded RNA PDB file and the SAXS fit files, along with the output from the SCOPER pipeline.

The SCOPER web app executes its calculations with the single best initial conformer out of the KGSRNA samples. The user can choose to optimize the excluded volume, hydration layer, or adjustment of offset in the SAXS fitting parameters. However, we recommend that the calculations be fixed with the excluded volume and hydration layer parameters to 1 for initial modeling. MultiFoXS is not used in the web app either, and only a single conformation with identified binding sites is returned as an output. We encourage users to report their results as such. More advanced users should be able to use our GitHub repository; however, they should be aware that, by not setting

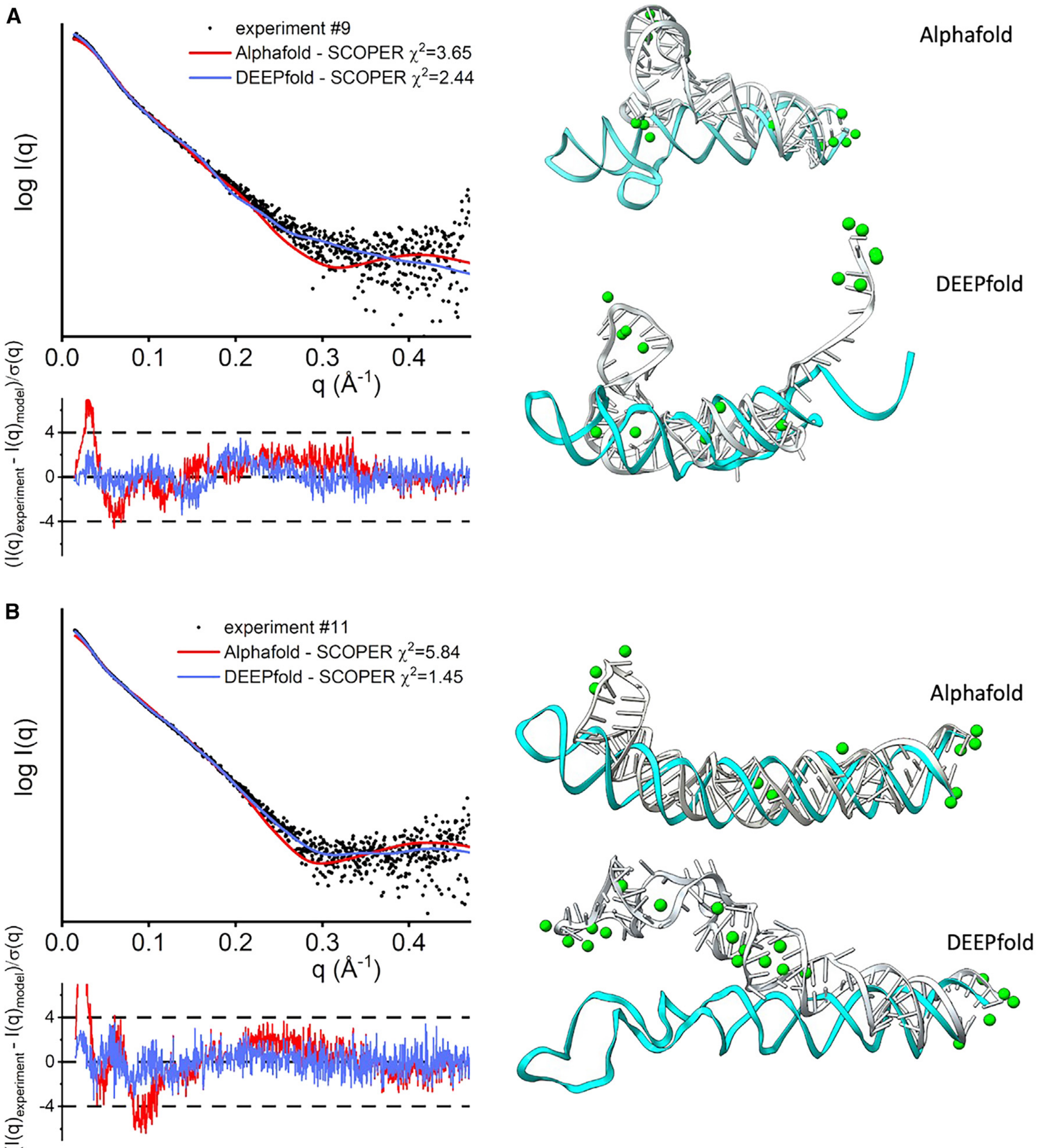


FIGURE 6 SCOPER results for benchmark cases #9 and #11 (labeled *A* and *B*). SAXS profile fit of AlphaFold3-refined structure (*red*), and DeepFoldRNA-refined structure with added $Mg^{2+}$ ions (*blue*) versus experimental profile (*black dots*), initial RNA structure (*cyan*) and best fitting RNA structure (*gray*) with predicted $Mg^{2+}$ ions (*green*) (*right column*).

the excluded volume and hydration layer parameters to 1 or using MultiFoXS, there is a possibility of overfitting the data.

## DISCUSSION AND CONCLUSION

Although there has been substantial progress in predicting RNA structures, different tools can generate widely varying models for the same RNA molecule. In such cases, the experimental SAXS profile serves as a valuable resource for validating or discarding these models. For this purpose, we have developed SCOPER, a novel pipeline that integrates a deep learning model for placing $Mg^{2+}$ ions into the RNA models or experimental structure, with SAXS-based validation to determine dynamic RNA conformation in solution. For ion placement, IonNet, a novel, deep-learning-based model was trained to predict $Mg^{2+}$ binding sites. Results show that these binding sites are predicted with high accuracy. Support for additional ions commonly found as structure stabilizers in the vicinity of RNA, such

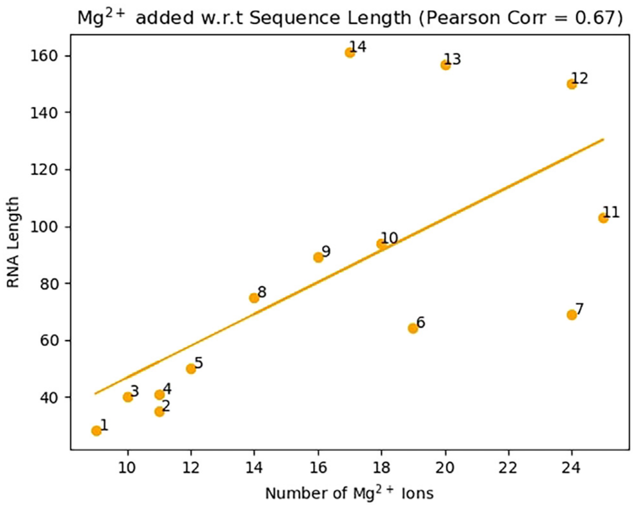


FIGURE 7 Number of $Mg^{2+}$ ions ($x$ axis) versus the sequence length ($y$ axis)
The correlation coefficient is 0.670.

as $Na^+$ and $K^+$, can be added with sufficient training data in future works.

The final ion positions and multistate models are selected based on a fit to SAXS data. However, data overfitting is possible when predictions of ions used in conjunction with experimental SAXS profiles select a subset of $Mg^{2+}$ ions that only improve the fit. To reduce overfitting, we recommend not varying adjustable SAXS fitting parameters, where adjusting the hydration layer may lead to the incorrect placement of $Mg^{2+}$. However, with only three SAXS data sets (*S*-adenosylmethionine riboswitch #10, P4P6 #13, and lysine riboswitch #14) that have an experimentally verified structure, we cannot be sure that our pipeline entirely prevents overfitting. We suggest using a high threshold (0.3–0.5 for our model) to obtain only the most confident IonNet predictions.

Another potential concern is our data set construction. Regarding the accuracy of each $Mg^{2+}$ ion position, errors commonly arise because they are isoelectronic with water and $Na^+$, leading to potential misidentification (59). By taking high-resolution structures, we attempt to mitigate this issue. We also leverage neural networks' ability to learn with noisy labels with large enough amounts of data (60,61). We assume our model could learn despite these possibly noisy labels due to the model having a high precision and low recall. This is because a low recall value but a high precision may indicate that the model could learn true binding motifs while false positive labels would have no such discernable pattern. IonNet can be improved further by explicitly considering $Mg^{2+}$ coordination when classifying binding sites. Most $Mg^{2+}$ ions are generally coordinated by oxygen and nitrogen atoms (62). In future work, we plan an additional data postprocessing step that classifies the coordination neighborhood based on distances to oxygen and nitrogen atoms. This classification can help improve the model's precision even further.

We show that an accurate RNA SAXS calculator relies on the presence of $Mg^{2+}$ ions in the model. The often-used extreme adjustment of the hydration layer, in the absence of $Mg^{2+}$ ions, can lead to the wrong selection of RNA conformations by SAXS fitting. Optimizing the predicted starting RNA structural model is crucial in delivering a good agreement with experimental SAXS profiles. Although the prediction of $Mg^{2+}$ ions in the RNA structure delivers a secondary improvement in the SAXS fit, it increases confidence in the RNA structure validation. This is particularly important when novel RNA structure prediction tools, such as AlphaFold3 (26), need to be validated. We also note that SCOPER is limited to validating or discarding structures and is highly dependent on the accuracy of the initial structural model.

As shown in our comparison of $\chi^2$ for all models sampled by KGSRNA with and without $Mg^{2+}$ (Fig. S5), adding the ions to the wrong model does not fit data better than the best conformer without $Mg^{2+}$. Also, using different folds of the same RNA (Fig. 6) shows that adding $Mg^{2+}$ to the wrong structure does not fit data better than more correct predictions. However, the accuracy of the modeling, whether it is selecting the conformer or placing the $Mg^{2+}$, also depends on data quality and the amount of RNA flexibility. Therefore the SCOPER pipeline should be considered a validation rather than a structure-determination tool in the relationship to SAXS data quality.

Overall, SCOPER's results prove that it can help find more accurate RNA structures and suggest their conformations that better represent the solution state. The fact that the multistate model did not significantly improve the SAXS fit in most cases suggests that accounting for the $Mg^{2+}$ in the RNA model is important to fit the experimental SAXS data properly. Here, we show that precisely calculated theoretical SAXS profiles can be used to validate or discard RNA structure predictions.

## DATA AND CODE AVAILABILITY

The SEC-SAXS were deposited to the SIMPLE SCATTERING database (simplescattering.com). In addition, the depositions contain the final merged SAXS curve and the final atomistic models used to calculate the SAXS fit (see Table S2). The RNA samples #4, #5, #7, #8, #9, #11, and #12 were collected under a proprietary agreement and unavailable in the SIMPLE SCATTERING database. The method is available from https://github.com/dina-lab3d/IonNet. For the latest release, please use our Zenodo link dina-lab3D/IonNet: Zenodo Release. A SCOPER pipeline is available as a web server from https://bilbomd.bl1231.als.lbl.gov/.

## ACKNOWLEDGMENTS

This research was partly supported by National Cancer Institute grant NCI P01 CA092584 to M.H., The U.S.-Israel Binational Science Foundation

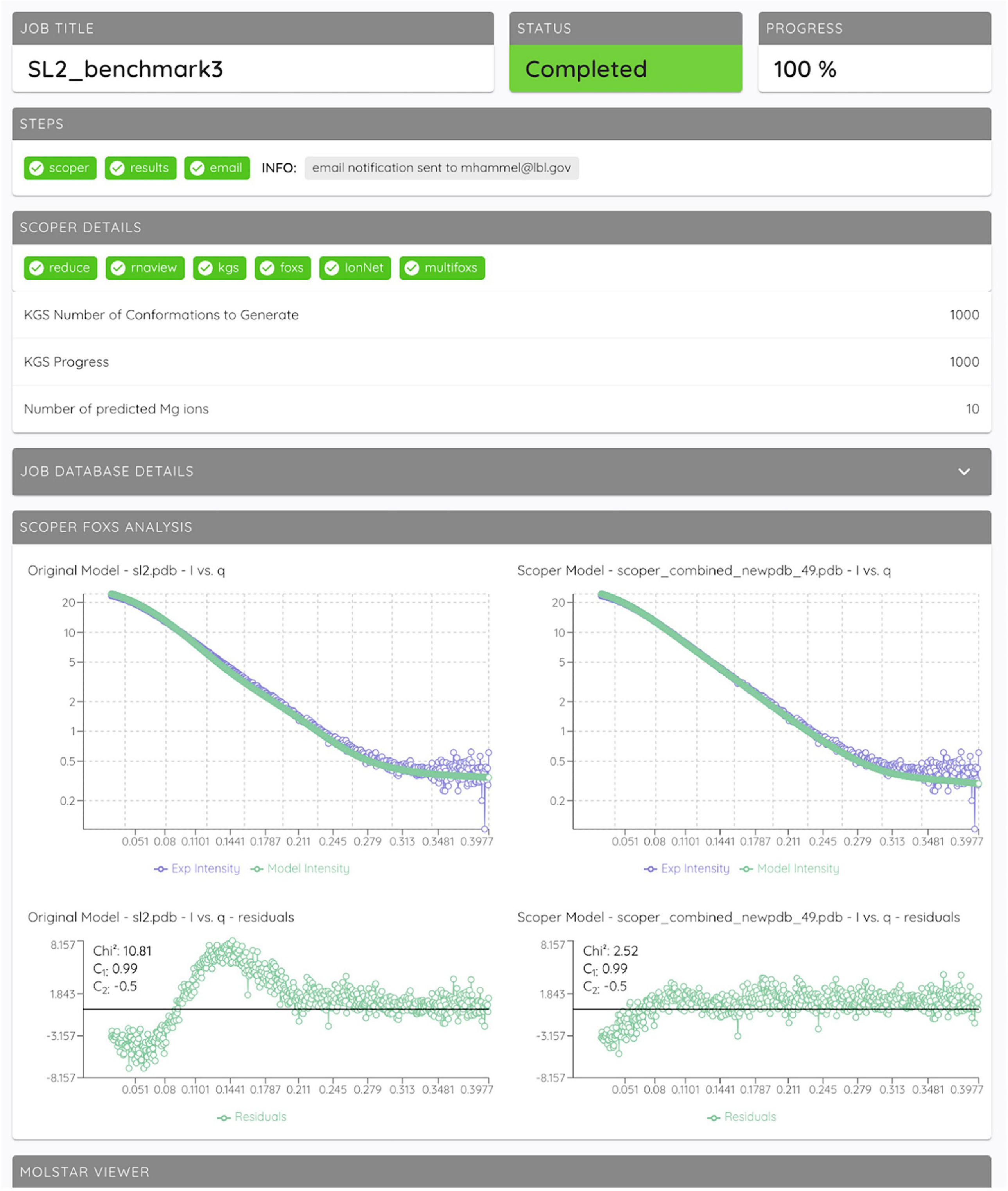


FIGURE 8 An example of the SCOPER job results page

(BSF) 2016070. SAXS data collection at SIBYLS is funded through NIGMS grant P30 GM124169-01, ALS-ENABLE, the IDAT program of the US Department of Energy Office of Biological and Environmental Research, and Biopreparednesss Research Virtual Environment (BRaVE) under contract no. DE-AC02-O5CH11231 and specifically the Taskforce 5 (DOE-BRAVET5) program supported by the U.S. Department of Energy,

Offices of Basic Energy Sciences. Molecular graphics and analyses performed with UCSF ChimeraX, developed by the Resource for Biocomputing, Visualization, and Informatics at the University of California, San Francisco, with support from National Institutes of Health R01-GM129325 and the Office of Cyber Infrastructure and Computational Biology, National Institute of Allergy and Infectious Diseases.

## AUTHOR CONTRIBUTIONS

D.S.-D. and M.H. designed the research. Data curation and SAXS experiments were performed by M.H. The methodology, software development, and benchmarking were carried out by E.P. Data analysis, visualization, and writing of the manuscript were performed by E.P., D.S.-D., and M.H. Writing of the paper was done by E.P., D.S.-D., and M.H. S.C. contributed to data analysis and web app development.

## DECLARATION OF INTERESTS

The authors declare no competing interests.

## SUPPORTING CITATIONS

References (62–67) appear in the supporting materiall.

## SUPPORTING MATERIAL

Supporting material can be found online at https://doi.org/10.1016/j.bpj.2024.12.024.

## REFERENCES


1. Mattick, J. S., and I. V. Makunin. 2006. Non-coding RNA. *Hum. Mol. Genet.* 15:R17–R29.
2. Esteller, M. 2011. Non-coding RNAs in human disease. *Nat. Rev. Genet.* 12:861–874.
3. Novikova, I. V., S. P. Hennelly, …, K. Y. Sanbonmatsu. 2013. Rise of the RNA Machines: Exploring the Structure of Long Non-Coding RNAs. *J. Mol. Biol.* 425:3731–3746.
4. Ma, H., X. Jia, …, Z. Su. 2022. Cryo-EM advances in RNA structure determination. *Signal Transduct. Targeted Ther.* 7:58.
5. Berman, H. M., J. Westbrook, …, P. E. Bourne. 2000. The Protein Data Bank. *Nucleic Acids Res.* 28:235–242.
6. Abramson, J., J. Adler, …, J. M. Jumper. 2024. Accurate structure prediction of biomolecular interactions with AlphaFold 3. *Nature.* 636:E4.
7. Crist, B. 1983. *In* A Review of: *"SMALL ANGLE X-RAY SCATTERING, 22*. O. Glatter and O. Kratky, eds. Academic Press, New York, pp. 377–378, Chemical Engineering Communications.
8. Feigin, L. A., Svergun D.I. 1987. Structure Analysis by Small-Angle X-Ray and Neutron Scattering, Vol. 1,. Plenum press, pp. 14–15.
9. Putnam, C. D., M. Hammel, …, J. A. Tainer. 2007. X-ray solution scattering (SAXS) combined with crystallography and computation: defining accurate macromolecular structures, conformations and assemblies in solution. *Q. Rev. Biophys.* 40:191–285.
10. Hammel, M. 2012. Validation of macromolecular flexibility in solution by small-angle X-ray scattering (SAXS). *Eur. Biophys. J.* 41:789–799.
11. Svergun, D. I., N. Burkhardt, …, K. H. Nierhaus. 1997. Solution scattering structural analysis of the 70 s Escherichia coli ribosome by contrast variation. II † . A model of the ribosome and its RNA at 3.5 nm resolution 1 †Paper I in this series is the accompanying paper, Svergun et al. (1997) 1Edited by M. F. Moody. *J. Mol. Biol.* 271:602–618.
12. Lipfert, J., and S. Doniach. 2007. Small-Angle X-Ray Scattering from RNA, Proteins, and Protein Complexes. *Annu. Rev. Biophys. Biomol. Struct.* 36:307–327.
13. Rambo, R. P., and J. A. Tainer. 2010. Improving small-angle X-ray scattering data for structural analyses of the RNA world. *RNA.* 16:638–646.
14. Schneidman-Duhovny, D., M. Hammel, …, A. Sali. 2013. Accurate SAXS profile computation and its assessment by contrast variation experiments. *Biophys. J.* 105:962–974.
15. Kirmizialtin, S., S. A. Pabit, …, R. Elber. 2012. RNA and Its Ionic Cloud: Solution Scattering Experiments and Atomically Detailed Simulations. *Biophys. J.* 102:819–828.
16. Chen, H., S. P. Meisburger, …, L. Pollack. 2012. Ionic strength-dependent persistence lengths of single-stranded RNA and DNA. *Proc. Natl. Acad. Sci. USA.* 109:799–804.
17. Drozdetski, A. V., I. S. Tolokh, …, A. V. Onufriev. 2016. Opposing Effects of Multivalent Ions on the Flexibility of DNA and RNA. *Phys. Rev. Lett.* 117:028101.
18. Chen, Y., and L. Pollack. 2016. SAXS studies of RNA: structures, dynamics, and interactions with partners. *Wiley Interdiscip. Rev. RNA.* 7:512–526.
19. Chen, Y.-L., T. Lee, …, L. Pollack. 2019. Conformations of an RNA Helix-Junction-Helix Construct Revealed by SAXS Refinement of MD Simulations. *Biophys. J.* 116:19–30.
20. Dethoff, E. A., J. Chugh, …, H. M. Al-Hashimi. 2012. Functional complexity and regulation through RNA dynamics. *Nature.* 482:322–330.
21. Thiel, B. C., G. Bussi, …, I. L. Hofacker. 2024. Sampling globally and locally correct RNA 3D structures using Ernwin, SPQR and experimental SAXS data. *Nucleic Acids Res.* 52:e73.
22. Chojnowski, G., R. Zaborowski, …, J. M. Bujnicki. 2023. RNA 3D structure modeling by fragment assembly with small-angle X-ray scattering restraints. *Bioinformatics.* 39:btad527.
23. Boniecki, M. J., G. Lach, …, J. M. Bujnicki. 2016. SimRNA: a coarse-grained method for RNA folding simulations and 3D structure prediction. *Nucleic Acids Res.* 44:e63.
24. Zacharias, M., and H. Sklenar. 2000. Conformational deformability of RNA: a harmonic mode analysis. *Biophys. J.* 78:2528–2542.
25. Sabei, A., T. G. Caldas Baia, …, E. Frezza. 2023. Internal normal mde analysis applied to RNA flexibility and conformational changes. *J. Chem. Inf. Model.* 63:2554–2572.
26. Fonseca, R., H. van den Bedem, and J. Bernauer. 2015. KGSrna: Efficient 3D Kinematics-Based Sampling for Nucleic Acids. *Lect. Notes Comput. Sci.* 80–95.
27. Philips, A., K. Milanowska, …, J. M. Bujnicki. 2012. MetalionRNA: computational predictor of metal-binding sites in RNA structures. *Bioinformatics.* 28:198–205.
28. Zhou, Y., and S.-J. Chen. 2022. Graph Deep Learning Locates Magnesium Ions in RNA. *QRB Discov.* 3:e20.
29. Manalastas-Cantos, K., P. V. Konarev, …, D. Franke. 2021. ATSAS 3.0: expanded functionality and new tools for small-angle scattering data analysis. *J. Appl. Crystallogr.* 54:343–355.
30. Battle, D. J., and J. A. Doudna. 2002. Specificity of RNA-RNA helix recognition. *Proc. Natl. Acad. Sci. USA.* 99:11676–11681.
31. Montange, R. K., and R. T. Batey. 2006. Structure of the S-adenosylmethionine riboswitch regulatory mRNA element. *Nature.* 441:1172–1175.
32. Garst, A. D., A. Héroux, …, R. T. Batey. 2008. Crystal structure of the lysine riboswitch regulatory mRNA element. *J. Biol. Chem.* 283:22347–22351.
33. Pearce, R., G. S. Omenn, and Y. Zhang. 2022. *De Novo* RNA tertiary structure prediction at atomic resolution using geometric potentials from deep learning. Preprint at BioRxiv. https://doi.org/10.1101/2022.05.15.491755.

34. Biesiada, M., K. J. Purzycka, …, R. W. Adamiak. 2016. Automated RNA 3D Structure Prediction with RNAComposer. *Methods Mol. Biol.* 1490:199–215.

35. Yang, H., F. Jossinet, …, E. Westhof. 2003. Tools for the automatic identification and classification of RNA base pairs. *Nucleic Acids Res.* 31:3450–3460.

36. Schneidman-Duhovny, D., M. Hammel, …, A. Sali. 2016. FoXS, FoXSDock and MultiFoXS: Single-state and multi-state structural modeling of proteins and their complexes based on SAXS profiles. *Nucleic Acids Res.* 44:W424–W429.

37. Veličković, P., G. Cucurull, …, Y. Bengio. 2017. Graph attention networks. *Preprint at arXiv.* https://doi.org/10.48550/arXiv.1710.10903.

38. Rossi, E., F. Monti, …, P. Liò. 2019. ncRNA classification with graph convolutional networks. *Preprint at arXiv.* https://doi.org/10.48550/arXiv.1905.06515.

39. Connolly, M. L. 1983. Analytical molecular surface calculation. *J. Appl. Crystallogr.* 16:548–558.

40. Schneidman-Duhovny, D., M. Hammel, and A. Sali. 2010. FoXS: a web server for rapid computation and fitting of SAXS profiles. *Nucleic Acids Res.* 38:W540–W544.

41. Rosenberg, D. J., G. L. Hura, and M. Hammel. 2022. Size exclusion chromatography coupled small angle X-ray scattering with tandem multiangle light scattering at the SIBYLS beamline. *Methods Enzymol.* 677:191–219.

42. Hopkins, J. B., R. E. Gillilan, and S. Skou. 2017. *BioXTAS RAW*: improvements to a free open-source program for small-angle X-ray scattering data reduction and analysis. *J. Appl. Crystallogr.* 50:1545–1553.

43. Meisburger, S. P., A. B. Taylor, …, N. Ando. 2016. Domain Movements upon Activation of Phenylalanine Hydroxylase Characterized by Crystallography and Chromatography-Coupled Small-Angle X-ray Scattering. *J. Am. Chem. Soc.* 138:6506–6516.

44. Semenyuk, A. V., and D. I. Svergun. 1991. GNOM – a program package for small-angle scattering data processing. *J. Appl. Crystallogr.* 24:537–540.

45. Rambo, R. P., and J. A. Tainer. 2013. Accurate assessment of mass, models and resolution by small-angle scattering. *Nature.* 496:477–481.

46. Svergun, D., C. Barberato, and M. H. J. Koch. 1995. CRYSOL– a program to evaluate X-ray solution scattering of biological macromolecules from atomic coordinates. *J. Appl. Crystallogr.* 28:768–773.

47. Liu, H., R. J. Morris, …, P. H. Zwart. 2012. Computation of small-angle scattering profiles with three-dimensional Zernike polynomials. *Acta Crystallogr. A.* 68:278–285.

48. Poitevin, F., H. Orland, …, M. Delarue. 2011. AquaSAXS: a web server for computation and fitting of SAXS profiles with non-uniformally hydrated atomic models. *Nucleic Acids Res.* 39:W184–W189.

49. Virtanen, J. J., L. Makowski, …, K. F. Freed. 2011. Modeling the hydration layer around proteins: applications to small- and wide-angle x-ray scattering. *Biophys. J.* 101:2061–2069.

50. Bai, Y., V. B. Chu, …, S. Doniach. 2008. Critical assessment of nucleic acid electrostatics via experimental and computational investigation of an unfolded state ensemble. *J. Am. Chem. Soc.* 130:12334–12341.

51. Bruetzel, L. K., T. Gerling, …, J. Lipfert. 2016. Conformational Changes and Flexibility of DNA Devices Observed by Small-Angle X-ray Scattering. *Nano Lett.* 16:4871–4879.

52. Qiu, X., K. Andresen, …, L. Pollack. 2007. Inter-DNA attraction mediated by divalent counterions. *Phys. Rev. Lett.* 99:038104.

53. Geller, K., and K. E. Reinert. 1980. Evidence for an increase of DNA contour length at low ionic strength. *Nucleic Acids Res.* 8:2807–2822.

54. Shliakhtenko, L. S., I. L. Liubchenko, …, V. B. Zhurkin. 1990. [The effect of temperature and ionic strength on the electrophoretic motility of synthetic DNA fragments]. *Mol. Biol.* 24:79–95.

55. 1999. DNA structure: cations in charge? *Curr. Opin. Struct. Biol.* 9:298–304.

56. Cate, J. H., A. R. Gooding, …, J. A. Doudna. 1996. Crystal structure of a group I ribozyme domain: principles of RNA packing. *Science.* 273:1678–1685.

57. Das, R., R. C. Kretsch, …, E. Westhof. 2023. Assessment of three-dimensional RNA structure prediction in CASP15. *Proteins.* 91:1747–1770.

58. Pelikan, M., G. L. Hura, and M. Hammel. 2009. Structure and flexibility within proteins as identified through small angle X-ray scattering. *Gen. Physiol. Biophys.* 28:174–189.

59. Leonarski, F., L. D'Ascenzo, and P. Auffinger. 2017. Mg2+ ions: do they bind to nucleobase nitrogens? *Nucleic Acids Res.* 45:987–1004.

60. Rolnick, D., A. Veit, …, N. Shavit. 2017. Deep learning is robust to massive label noise. Preprint at arXiv. https://doi.org/10.48550/arXiv.1705.10694.

61. Song, H., M. Kim, …, J.-G. Lee. 2023. Learning From Noisy Labels With Deep Neural Networks: A Survey. *IEEE Transact. Neural Networks Learn. Syst.* 34:8135–8153.

62. Zheng, H., I. G. Shabalin, …, W. Minor. 2015. Magnesium-binding architectures in RNA crystal structures: validation, binding preferences, classification and motif detection. *Nucleic Acids Res.* 43:3789–3801.

63. Fout, A., J. Byrd, A. Ben-Hur…, 2017. Protein Interface Prediction using Graph Convolutional Networks. *Adv. Neural Inf. Process. Syst.* 30:6533–6542.

64. Vinyals, O., S. Bengio, and M. Kudlur. 2015. Order Matters: Sequence to sequence for sets. Preprint at arXiv. https://doi.org/10.48550/arXiv.1511.06391.

65. Huang, L., J. Wang, …, D. M. J. Lilley. 2017. Structure of the Guanidine III Riboswitch. *Cell Chem. Biol.* 24:1407–1415.e2.

66. Liu, Y., D. Esyunina, …, D. J. Patel. 2018. Accommodation of Helical Imperfections in Rhodobacter sphaeroides Argonaute Ternary Complexes with Guide RNA and Target DNA. *Cell Rep.* 24:453–462.

67. Huang, L., J. Wang, and D. M. J. Lilley. 2020. Structure and ligand binding of the ADP-binding domain of the NAD riboswitch. *RNA.* 26:878–887.

Biophysical Journal, Volume *124*

## Supplemental information

# Predicting RNA structure and dynamics with deep learning and solution scattering

**Edan Patt, Scott Classen, Michal Hammel, and Dina Schneidman-Duhovny**

**Predicting RNA Structure and Dynamics with Deep Learning and Solution Scattering**

Edan Patt[1], Scott Classen[2], Michal Hammel[2*], Dina Schneidman-Duhovny[1*]

[1]School of Computer Science and Engineering, The Hebrew University of Jerusalem, Israel
[2]Molecular Biophysics and Integrated Bioimaging, Lawrence Berkeley National Laboratory, Berkeley, CA, USA.

## Supplementary Material

### 1. IonNet Training and Metrics.

**Data generation.** We downloaded 1,407 PDB format files from the Protein Data Bank (PDB) (1) that contain RNA and $Mg^{2+}$ atoms with a resolution below 3Å. We extracted all $Mg^{2+}$ ions within 8Å from the RNA molecule for each structure. These ions serve as our positive samples. We also randomly selected water molecules within 8Å from the RNA molecule to serve as our negative samples. We selected samples with a ratio of 1.5 water molecules per one $Mg^{2+}$ ion. In total, 41,725 $Mg^{2+}$ ions and 65,482 water molecules were selected. We augmented this dataset by generating additional $Mg^{2+}$ and water positions with the purpose of reducing sensitivity to the accuracy of the positions during the inference stage. To generate these positions, we relied on the probe centers of the solvent-accessible surface (2). The probe was placed at 1.4Å from the RNA surface, which we found to be the average distance for most $Mg^{2+}$ ions from the RNA in our dataset (Fig. S6). The density of the probes was set to 1 probe per $Å^2$ to optimize the trade-off between the accuracy of the probe position and the runtime performance of the inference stage. Probes were labeled as positive if they were at a distance of 3Å from an existing $Mg^{2+}$ ion and negative otherwise. In total 273,900 such probes were added to the dataset, with ~90,000 positive samples and the rest being negative samples.

**Data separation into train, validation, and test sets.** To construct non-overlapping train, validation, and test sets, we ran an exhaustive pairwise sequence alignment between all the RNA chains in our dataset. The dataset construction consisted of three main stages. First, we removed all sequences that had high sequence identity to many other sequences. Second, we detected similar pairs of sequences to prevent them from being separated into the train and test sets. Third, we divided the remaining sequences into the train, test, and validation sets in accordance with the marked pairs from the second stage.

In the first stage, we calculated an average sequence identity to all other sequences. This enabled us to discard very short or very long sequences that had high sequence identity to a large fraction of other sequences in the dataset. A sequence was discarded if the average sequence identity was above 56%, resulting in a more divergent selection of sequences, as seen in the similarity matrix (Fig. S7.). This filtering resulted in 961 sequences down from the original 1,538 RNA sequences. Measuring sequence identity scores between pairs of sequences with significantly different lengths results in different score distributions compared to score distributions measured between sequences of similar lengths. For this reason, we needed to obtain the distribution of sequence identity scores between sequences of differing lengths.

To solve this problem, in the second stage, all remaining sequences were placed into bins in accordance with their length. Small-sized bins were merged together with larger bins of similar lengths. We ended up with nine bins with a similar number of RNA chains (Table S3). Similar pairs were banned from being separated into the train and test sets if either their sequence identity was above 90% or if their sequence identity scores were in the top 1% in the score distribution between all pairs in both bins.

In the third stage, we randomly add 5% of the 961 filtered sequences into our test set. All the chains derived from that PDB structure are also included in the test set. For each of the remaining 95% of sequences we compared each potential chain to the RNA sequences in the test set. If the sequence was not banned from the training set for being too similar to a sequence in the test set, then it was added to the training set. Otherwise, it was added to the validation set. In total our best performing model used 580 chains for training, 91 for validation and 47 for testing. These come from 499 PDB files, 447 solved with X-ray, 49 with EM and 3 with NMR. This corresponds to 19,855 $Mg^{2+}$ ion positions for training, 3324 for validation and 638 for testing. With negative samples and augmented data included, train data used 45,753 positive samples and 113,941 negative samples, validation used 5770 positive and 18,628 negatives and the test set consisted of 1371 positives and 3584 negatives. To our knowledge these are the largest training and test sets used to train and evaluate models of a similar nature to IonNet.

**Problem definition.** The goal of our model is to take in as input an embedding of a neighborhood of either $Mg^{2+}$, water molecules, or surface probes near RNA atoms. We define the neighborhood of the ion, water molecule or probe as all atoms in a 8Å radius. The model then must label the neighborhood embedding of $Mg^{2+}$ or probes near (up to 3Å) to such an ion as positive and neighborhoods of water or probes far away (further than 3Å) from $Mg^{2+}$ ions as negative. For our models, we experimented with two main architectures: 3D-Convolutional Neural Networks (3D CNNs) and Graph Neural Networks (GNNs). Our embedding functions either embed neighborhoods into a 3D voxelized grid or into a graph where every node represents a single atom. The convolutional models proved ineffective, so in this work, we only discuss the training process and metrics for the GNNs and compare the results to the 3D CNN architectures.

**Feature definition.** Each node in the graph received a vector embedding of the physicochemical properties of each RNA atom. These atoms are characterized using the following 12 atom types according to SYBYL mol2 definition: carbon (C.2, C.3 and C.ar), nitrogen (N.2, N.4, N.am, N.ar, and N.p13), oxygen (O.2, O.3 and O.co2), phosphate (P.3) and sulfur (S.3). Additional features include the solvent accessible area of the atom (ASA) and its partial charge. In the embedding vector, the 13 first channels were used as a one hot encoding vector for the types of elements that were possibly used according to the SYBYL Mol2 format. An additional channel was used to label $Mg^{2+}$ ions or water molecules.

**GNN architecture**. As many natural things, such as networks, drug-like molecules or larger biomolecules, can be represented as graphs, deep learning applications using GNNs had a significant impact (3). Graphs have an inherent advantage over 3D CNNs, because of their compact representation compared to the sparse representations of 3D CNNs. Moreover, graph-based representations are invariant to rotations and translations, making them a much more natural representation for molecules, as the

orientation of the molecule should not affect the data's representation. To represent a neighborhood near $Mg^{2+}$ ion or a water molecule, we represented all the heavy atoms as nodes in a graph. The first node in the graph is always one of these two molecules. Molecules were selected only if they were within 8Å of the RNA atoms. Two nodes are considered connected within the graph if they are at most 8Å apart. In addition to the vector embedding described in the Feature Definition section, each edge also holds the distance between the two atoms. Two main architectures were used in our experiments: graph convolutional networks(4) and graph attention networks (5). Our graph convolutional model consists of multiple graph convolutions followed by batch normalization. The classifier head consists of a global pooling layer named set2set (6) for aggregating the node representations, followed by two linear layers. Our graph attention model uses three attention layers with 12,11,4 heads respectively in each layer. Each graph attention layer is followed by a batch normalization layer. A classification head of two fully connected layers is then used at the end for the classification. A final attempt at creating a model using GNNs was made by combining the two concepts together. First, running the inputs through a few graph attention layers and only then running a convolutional network. As our most successful model used the combination of both types of graph neural networks (Fig. S1).

**Training.** As mentioned in the preprocessing section, an inherent bias was added toward negative samples. This was done purposefully to make our model more sensitive towards positive samples rather than negative samples. To deal with this bias, during training, we used a binary cross entropy loss weighted with a higher weight for positive samples. The loss can be written as such:

$$WeightedBCELoss = -(ylog(p)a + (1 - y)log(1 - p))$$

Where y is the label, p is the prediction, and a is the positive weight multiplied by the positive label loss to combat the positive and negative sample imbalance. We found that $a \sim 1.8$ worked best for us. All models were trained with either Adam or AdamW optimizers depending on whether or not the model tended to overfit the training data with a learning rate of around 0.0001. Another aspect during training was data augmentation. We tried to add random Gaussian noise to the location of $Mg^{2+}$ ion graphs. This was done to simulate probes in the vicinity of the ion. The data was augmented during training by moving the coordinate of the ion slightly and then computing the distances to the neighboring nodes. The augmentation ensured that no impossible graphs would be created, meaning the $Mg^{2+}$ ion would never be moved too close or too far from one of its neighbors. To our surprise, the model was able to pick up that this noise was being added only to positive samples. We concluded that this noise was interfering with the distribution of some geometric properties of the location of the ion relative to its neighbors.

**Inference.** To generate potential probe positions next to the RNA surface, we calculated the Connolly solvent accessible surface with a probe radius of 1.4Å and density of 0.5 dots per Å. Probe centers are used as potential $Mg^{2+}$ sites. During the inference stage, the model goes over each probe position, creates an embedding of the probe's neighborhood, and predicts whether or not it is a potential $Mg^{2+}$ binding site. To combat the phenomena of many close-together probes near a potential binding site being labeled as positive by the model, we use iterative clustering to select the probe with the highest confidence within a 1.5Å radius.

**IonNet metrics.** In addition to standard metrics for model performance, we also evaluate the accuracy of the predicted positions by our inference pipeline. We define the Distance Center Center metric (DCC) based on the distance between the center of the predicted binding site to the center of the actual binding site as follows:

$$DCC = \frac{\#ions\ with\ positive\ probes\ within \leq 4\text{Å}}{\#of\ ions}$$

**MetalIonRNA comparison**. MetalIonRNA (7) relied on 50 RNA structures, containing 175 $Mg^{2+}$ ions using 5-fold cross-validation. Because this amount of ions is relatively small for training neural networks, we sampled probes (see Inference above) and added them to the dataset. Probes within 0.72Å from experimentally known binding sites of $Mg^{2+}$ were given positive labels, and the rest were given negative labels. We ended up with a training set of 735 positive samples and 39,065 negative samples and a test set with 229 positive samples and 13,571 negative samples in one of our splits. To deal with this data imbalance, we used a random weighted sampler. In our comparison, we reproduced the MetalIonRNA experiment as closely as possible by training our own model on this dataset of RNA structures.

## 2. IonNet performance

**Architectures and model accuracy.** We tested several network architectures, including 3D-CNN, and GNNs with graph attention layers, graph convolution layers, and a combination of both layers (Table S1). 3D-CNN had the worst performance and the longest training time (days on a single GPU) due to suboptimal data representation. GNN-based architectures had a significantly better performance, with the best results achieved by the combination of convolution and attention layers with a training time of a few hours. We believe the reason using attention and convolutional layers together works best is because the attention layers learn intricate connections between nodes, improving the embeddings. Convolutions on the other hand, enforce an inductive bias that, we believe, increases the importance of close-together nodes as multiplication is applied using the distance between neighboring nodes. Four-fold cross-validation was performed with our best-performing model resulting in an even more precise AUROC estimate for the model with a mean of 0.89 (Fig. 2A). We provide the size of each such fold (Table S4, Table S5.)

**ASA is the most important feature for classification.** We performed an ablation study to identify features that contributed most to the model's performance. We removed the charge, ASA, or atom type (Feature definition) for every one of the nodes in the graph by setting their values to zero. We found that the ASA had the most significant contribution to the model's accuracy, followed by the atom type, while the charge had little to no effect (Fig. S8A). We assume that the model focuses on the geometric properties of the structure, more so than the physicochemical ones. ASA values provide information about the shape of the ion neighborhood. Most likely, $Mg^{2+}$ ions are fitted in cavities with sufficient space for them and surrounding waters (Fig. S9). We attribute this to the geometric shape information present in the graph neural network representation. We also assume that the charge information has little contribution because it is redundant due to the atom type.

**Comparison to MetalIonRNA.** MetalIonRNA is a statistical approach for placing ions based on their distances to the N or an O atom pairs. We trained IonNet using the structures from the MetalIonRNA

$Mg^{2+}$ dataset. Despite the low number of structures, our model had an average AUROC of 83.3% (Fig. S8.)

**Comparison to MgNet.** MgNet (8) is a 3D convolutional neural network for finding $Mg^{2+}$ binding sites in RNA structures. MgNet's reported metrics over the test set is an average recall of 46.9 and an average precision of 35.5 over all of test set folds, which is significantly lower compared to IonNet. (Fig. 2A, Table S1.)

**Inference scoring with DCC.** Our inference process employs iterative clustering to identify $Mg^{2+}$ positions with the highest confidence, according to IonNet. Subsequently, we evaluated the DCC score on the model's prediction following the iterative clustering step on the $Mg^{2+}$ of our test set. The resulting weighted average DCC score of 0.46 is comparable to the 0.51 recall score exhibited by our model on the test set prior to filtering. This demonstrates that the model's confidence is an effective way to select a subset of $Mg^{2+}$ ion positions without sacrificing much of the model's predictive capabilities (Table S1).
Additionally, we present the distribution of DCC scores across our test set (Fig. S10). Furthermore, we illustrate three examples of our inference outcomes from our test set that are indicative of the model's performance: one exemplary result, one average result, and one poor performing result. These examples were selected from high-resolution structures where the confidence in $Mg^{2+}$ positions is high. These structures are 5nz3 (9) with a resolution of 2.059, 6d8f (10) with a resolution of 2.15 and 6tf2 (11) with a resolution of 2.55. For each structure, we measured a DCC score of 0.6, 1.0, and 0.2, respectively. When increasing the model's confidence threshold for a positive sample from 0.5 to 0.9 the model's DCC on these examples did not decrease (Fig. S11).

**IonNet inference vs Random Selection**:
In Fig. 2 B-D we give an example of IonNet's inference capabilities. Here we expand upon this example and explain the probability of randomly achieving such a result.
In our example (Fig. 2 B-D) there are 5,668 probes, of which only 111 are within a 3Å radius of any $Mg^{2+}$ ion (there are 12 $Mg^{2+}$ ions). The probability of selecting 30 such probes randomly and having none of them within a 3Å radius of any Mg ion is:

$$P(0) = \frac{\left(\frac{5557}{30}\right)}{\left(\frac{5668}{30}\right)} = 54\%$$

Where $\left(\frac{a}{b}\right)$ denotes the combinatorics "choose" operation.
A general* formula for the probability of 'n' correct guesses is

$$P(n) = \frac{\left(\frac{111}{n}\right)*\left(\frac{5557}{30-n}\right)}{\left(\frac{5668}{30}\right)}$$

The expected value is thus.

$$E[correct\ guesses] = \Sigma_{n=0}^{12} P(n) * n \approx 1.58$$

*Note that this is an upper bound on the probability and the expectation since, in this oversimplified calculation, we assume no two probes share the same $Mg^{2+}$ ion as a neighbor

On average, when selecting 30 random probes in this example, we would have only 1.58 good guesses. Whereas in our results, we found 6 such probes, the probability for a random selection such as this is ~0.000018.

### 3. Data collection SEC-MALS

Eluent was subsequently in line with a series of UV at 280nm, MALS, quasi-elastic light scattering (QELS), and refractometer detector. MALS experiments were performed using an 18-angle DAWN HELEOS II light scattering detector connected in tandem with an Optilab refractive index concentration detector (Wyatt Technology). System normalization and calibration were performed with bovine serum albumin using a 55 µL sample at 7 mg/mL in the same SEC running buffer, and a dn/dc value calibrated by BSAS was used to further determine MW by MALS. The light scattering experiments were used to perform analytical scale chromatographic separations for MW determination of the principal peaks in the SEC analysis. UV, MALS, and differential refractive index data were analyzed using Wyatt Astra 7 software to additionally monitor the homogeneity of the sample across the elution peak.

**Supporting References:**


1. Berman,H.M. (2000) The Protein Data Bank. *Nucleic Acids Research*, **28**, 235–242.

2. Connolly,M.L. (1983) Analytical molecular surface calculation. *Journal of Applied Crystallography*, **16**, 548–558.

3. Fout,A., Byrd,J., Shariat,B. and Ben-Hur,A. (2017) Protein Interface Prediction using Graph Convolutional Networks. *Adv. Neural Inf. Process. Syst.*, **30**.

4. Rossi,E., Monti,F., Bronstein,M. and Liò,P. (2019) ncRNA Classification with Graph Convolutional Networks. 10.48550/arXiv.1905.06515.

5. Veličković,P., Cucurull,G., Casanova,A., Romero,A., Liò,P. and Bengio,Y. (2017) Graph Attention Networks. 10.48550/arXiv.1710.10903.

6. Vinyals,O., Bengio,S. and Kudlur,M. (2015) Order Matters: Sequence to sequence for sets. 10.48550/arXiv.1511.06391.

7. Philips,A., Milanowska,K., Lach,G., Boniecki,M., Rother,K. and Bujnicki,J.M. (2012) MetalionRNA: computational predictor of metal-binding sites in RNA structures. *Bioinformatics*, **28**, 198–205.

8. Zhou,Y. and Chen,S.-J. (2022) Graph deep learning locates magnesium ions in RNA. *QRB Discov*, **3**.

9. Huang,L., Wang,J., Wilson,T.J. and Lilley,D.M.J. (2017) Structure of the Guanidine III Riboswitch. *Cell Chem Biol*, **24**, 1407–1415.e2.

10. Liu,Y., Esyunina,D., Olovnikov,I., Teplova,M., Kulbachinskiy,A., Aravin,A.A. and Patel,D.J. (2018) Accommodation of Helical Imperfections in Rhodobacter sphaeroides Argonaute Ternary Complexes with Guide RNA and Target DNA. *Cell Rep.*, **24**, 453–462.

11. Huang,L., Wang,J. and Lilley,D.M.J. (2020) Structure and ligand binding of the ADP-binding domain of the NAD riboswitch. *RNA*, **26**, 878–887.

| Model Type | AUROC ↑ | Precision ↑ | Recall ↑ | Specificity ↑ |
|---|---|---|---|---|
| 3D-CNN | 0.73 | 0.68 | 0.40 | 0.90 |
| Graph Attention | 0.86 | 0.79 | 0.50 | 0.82 |
| Graph Convolution | 0.81 | 0.55 | 0.73 | 0.77 |
| Graph Attention + Convolution | 0.88 | 0.82 | 0.51 | 0.96 |

**Table S1.** Network architectures and their performance over all data from the test set, 1371 positive samples and 3584 negative samples

| Sample | SIMPLE SAXS ID | Length (# nucleotide) | Buffer | MW Seq (kDa) | MW SAXS (kDa) | MW MALS (kDa) | Dmax (Å) | Rg (Å) |
|---|---|---|---|---|---|---|---|---|
| #1 | XSEF1RT7 | 28 | 10mM HEPES pH 7.5 100mM KCl, 5mM MgCl2, 1mM TCEP | 8.9 | 10 | ND | 57 | 15.9 |
| #2 | XSSMOQZI | 35 | 10mM HEPES pH 7.5 100mM KCl, 5mM MgCl2, 1mM TCEP | 11.2 | 12 | ND | 60 | 17.4 |
| #3 | XSEFXJZF | 40 | 20mM Tris pH 7.4, 150mM KCl, 5mM MgCl2 | 12.9 | 12 | 12 | 65 | 17.5 |
| #4 | ND | 41 | 20 mM BisTris pH 6.5, 100mM KCl, 2mM MgCl2 | 13.4 | 14 | 19 | 74 | 21.6 |
| #5 | ND | 50 | 20mM Hepes pH 7.4, 100mM KCl, 3mM MgCl2 | 16.0 | 16 | ND | 70 | 21.4 |
| #6 | XSPSCH50 | 66 | 20mM Hepes, 150 mM NaCl, 1 mM TCEP pH 7.5, 2mM Mg Cl2 | 20.5 | 20 | 20 | 108 | 25.8 |
| #7 | ND | 69 | 20 mM BisTris pH 6.5, 100mM KCl, 2mM MgCl2 | 22.3 | 21.4 | ND | 84 | 24.4 |
| #8 | ND | 76 | 20mM Hepes pH 7.4, 100mM KCl, 3mM MgCl2 | 25.2 | 31 | 34 | 100 | 29.6 |
| #9 | ND | 90 | 20mM Hepes pH 7.4, 100mM KCl, 3mM MgCl2 | 29.5 | 33 | ND | 115 | 33.4 |
| #10 | XSIEWNFS | 94 | 20 mM MOPS at pH 6.5, 50 mM KCl, and 7.6 mM $MgCl_2$ + SAM I | 29.1 | 32 | 29.9 | 81 | 22.4 |
| #11 | ND | 111 | 20mM Hepes pH 7.4, 100mM KCl, 3mM MgCl2 | 36.0 | 35.1 | 40 | 160 | 41.1 |
| #12 | ND | 150 | 20mM Hepes pH 7.4, 100mM KCl, 3mM MgCl2 | 48.9 | 42 | 50 | 170 | 41.8 |
| #13 | XSHPX9SN | 160 | 20mM Tris pH 7.4, 300mM KCl, 5mM MgCl2 | 51.8 | 50 | 56 | 108 | 29.6 |
| #14 | XSKSZRJZ | 161 | 20 mM HEPES at pH 6.5, 50 mM KCl, and either 5 mM $MgCl_2$,+2 mM lysine | 49.9 | 47.6 | ND | 108 | 31.1 |

**Table S2.** Experimental SAXS and MALS parameters

| Bin Lengths | Bin Size |
|---|---|
| (0,20) | 130 |
| (20,30) | 112 |
| (30,55) | 110 |
| (55,70) | 97 |
| (70,80) | 92 |
| (80,100) | 102 |
| (100,125) | 149 |
| (125,415) | 91 |
| (450, 2855) | 77 |

**Table S3.** Preprocessing Sequence Bin Sizes

| Dataset | Train Size | Validation Size | Test Size |
|---|---|---|---|
| Fold 1 | 16,482 | 2,445 | 1,123 |
| Fold 2 | 16,601 | 2,334 | 1,062 |
| Fold 3 | 19,855 | 3,324 | 638 |
| Fold 4 | 21,659 | 1,047 | 804 |

**Table S4.** Amount of Mg ion binding sites used in each fold, these numbers do not include the added augmented ion positions later added to increase the size of the dataset.

| Dataset | Positive Samples | Negative Samples |
|---|---|---|
| Fold 1 | 3,177 | 6,405 |
| Fold 2 | 2,869 | 6,977 |
| Fold 3 | 1,371 | 3,584 |
| Fold 4 | 2,147 | 4,342 |

**Table S5.** Amount of positive and negative samples in each test set, this includes the additional augmented data that increases the size of the dataset.

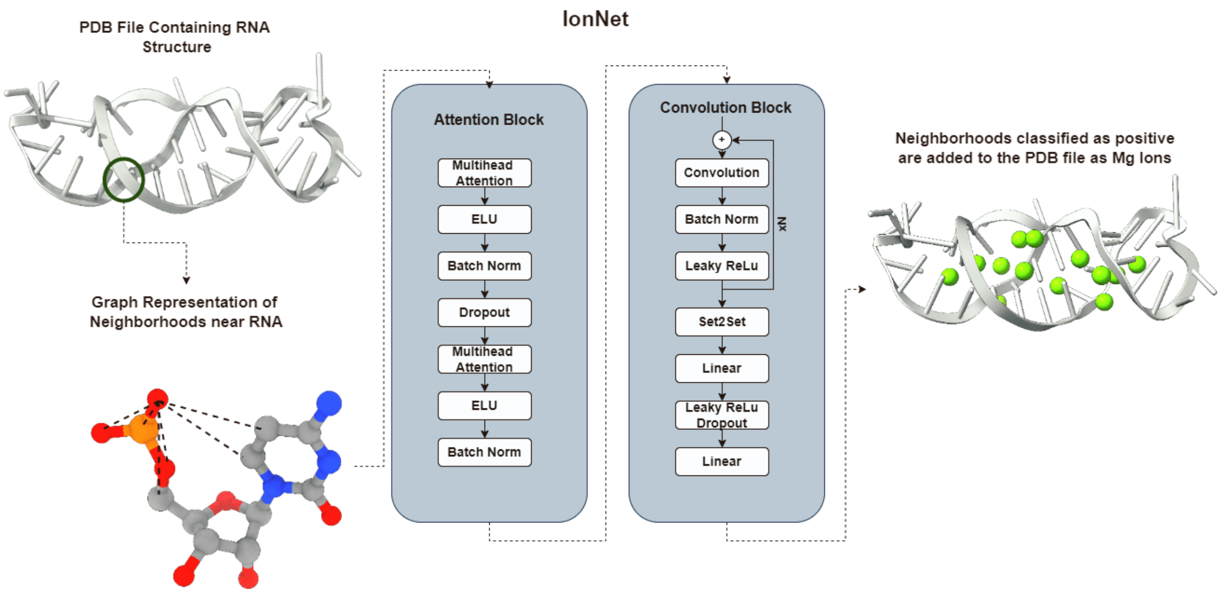


**Figure S1**. Our best performing model architecture, consisting of both graph convolution and graph attention layers.

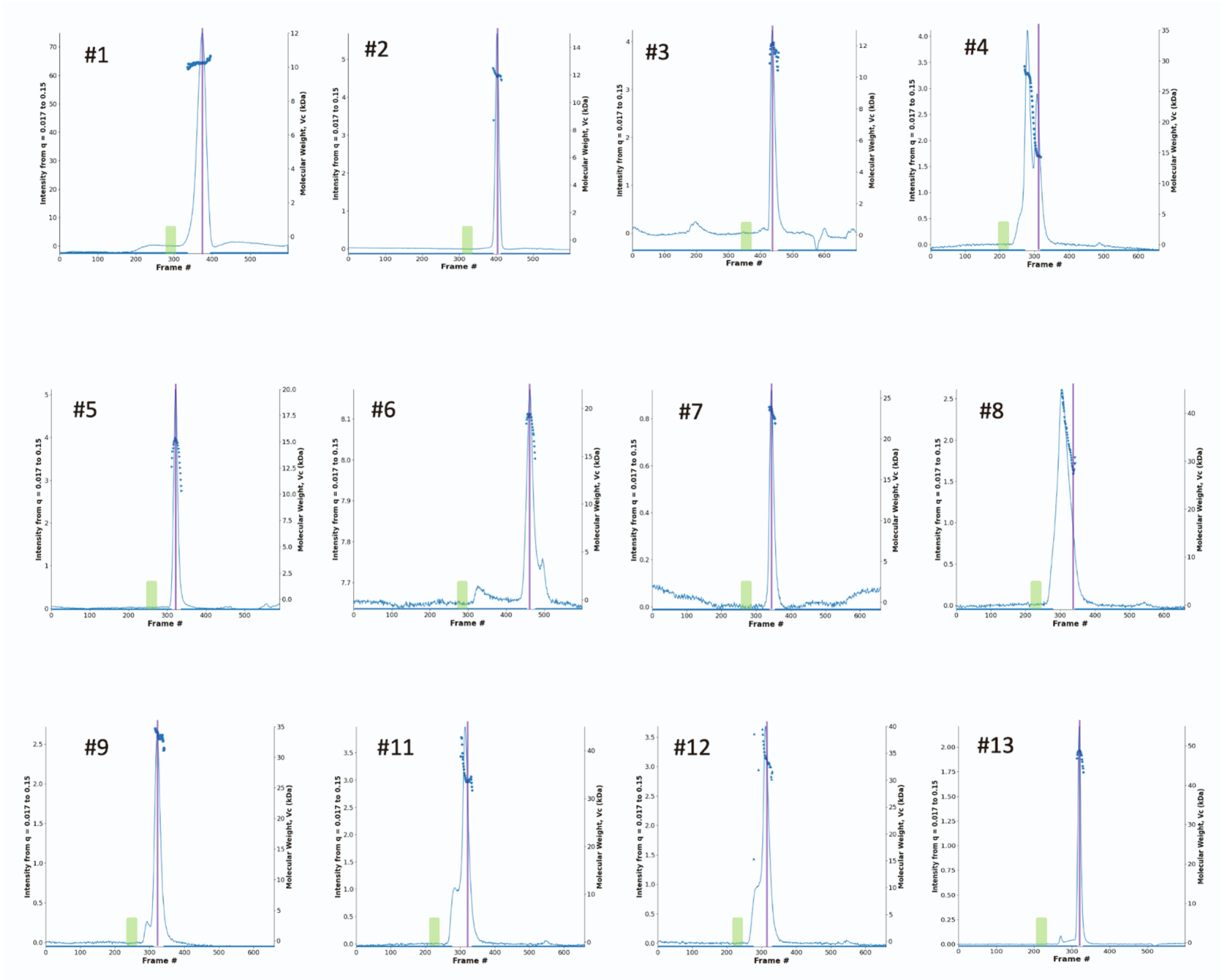


**Figure S2.** The SEC-SAXS chromatograms for each RNA sample show integrated SAXS intensity in the *q* range of 0.017- 0.17 vs. frame (line), and, if available, Molecular weight vs. frame (symbols). Green-shaded regions are buffer regions, and purple-shaded regions are sample regions. RNA #10 and #14 were collected at the SIBYLS beamline previously and reported in (1,2)

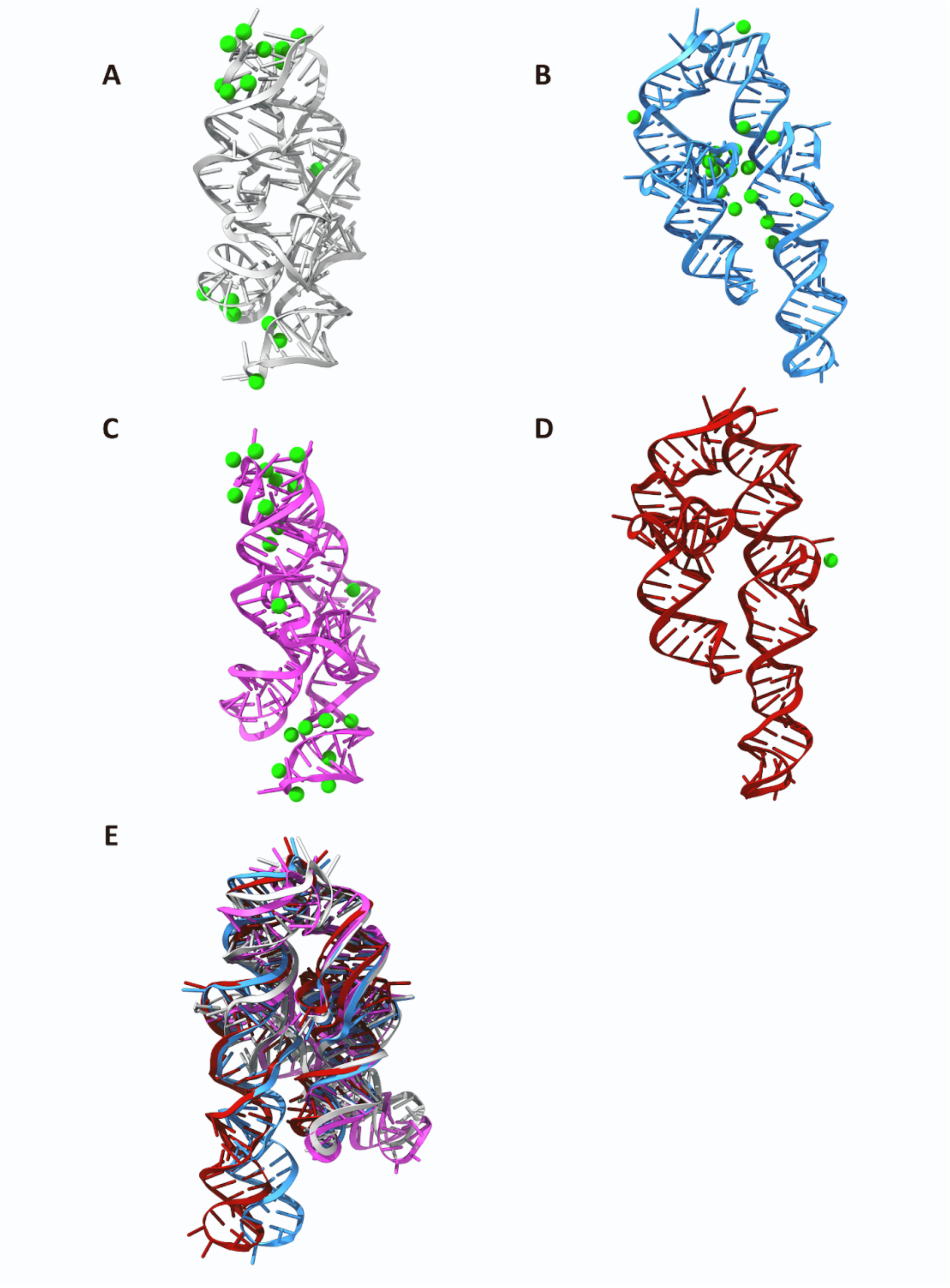


**Figure S3. Multi-state fitting of P4P6 (sample #13)**. A,B,C,D Four conformations were selected, along with their respective ions, by the MultiFoXS program with the weights of 0.084, 0.337, 0.522, and 0.057, respectively. E. Structural alignment of the four conformations using ChimeraX.

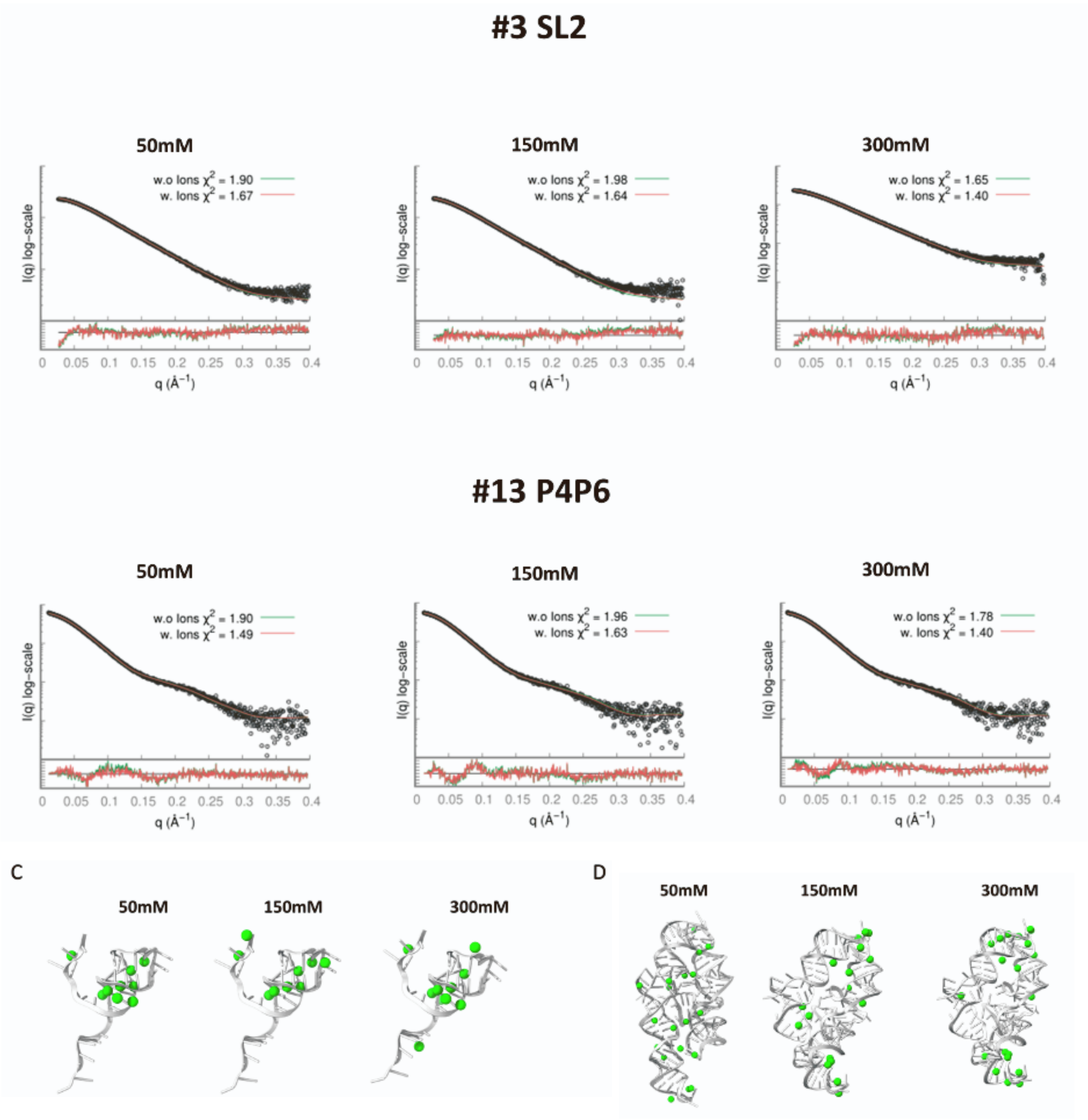


**Figure S4. (A-B)** Experimental SAXS curves (black) for samples #3 and #13 in comparison for the best fit - structure without $Mg^{2+}$ ions and unconstrained c1 and c2 values (green curve). The selected best-fit structure with added $Mg^{2+}$ ions that were selected by running a combinatorial SAXS-based selection of ions while unconstrained. **C-D.** #3 (RNA stem-loop) and #13 (P4P6) structures, respectively. In each figure, we see how ionic strength affects the placement of $Mg^{2+}$ ions (green). The stem-loop structure remains the same across all concentrations, while the selected P4P6's structure at the 50mM KCl shows small differences in the compaction relative to 150mM and 300mM conditions

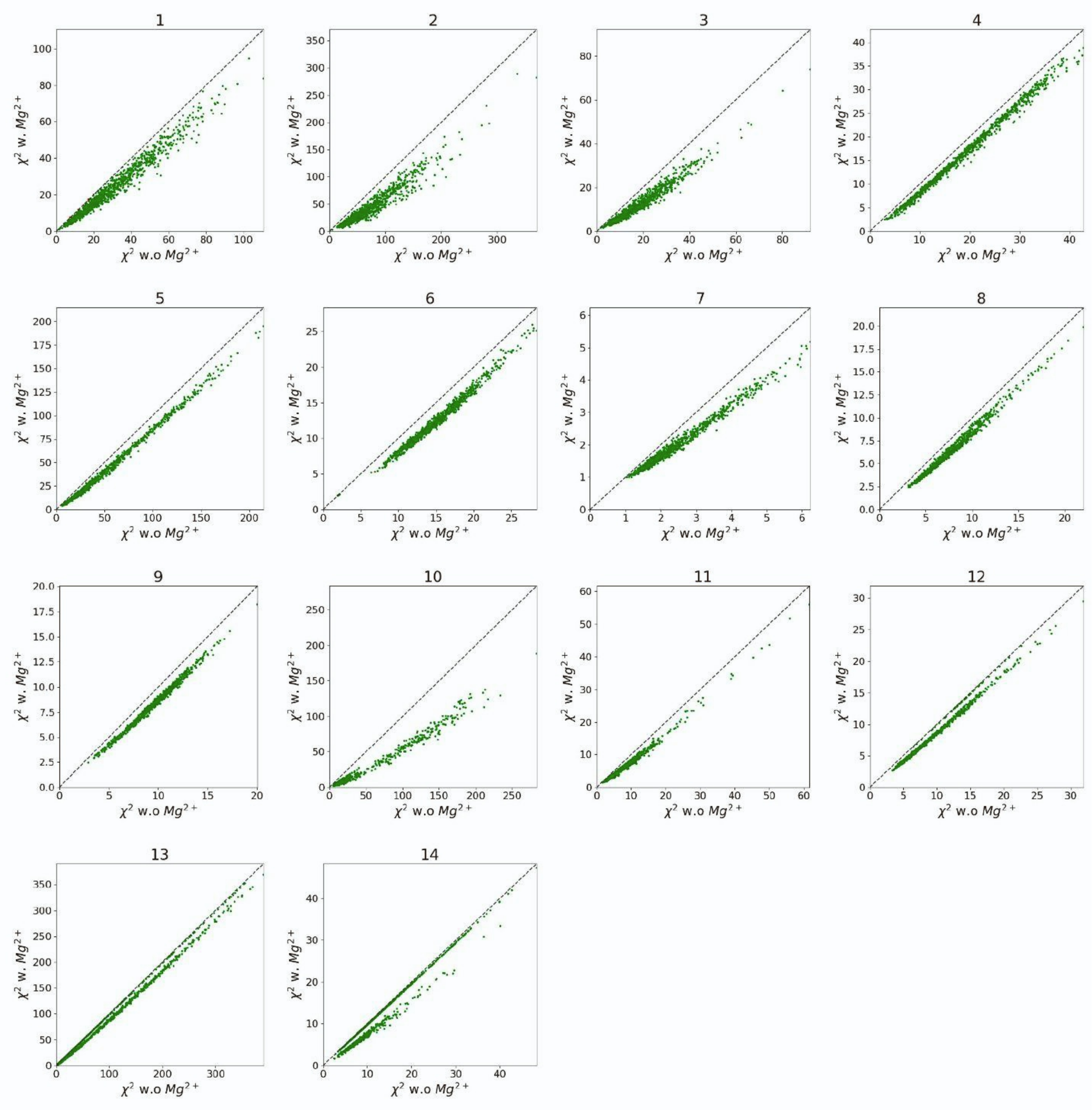

**Figure S5.** $\chi 2$ before and after adding $Mg^{2+}$ ions to all structures sampled by KGSRNA.

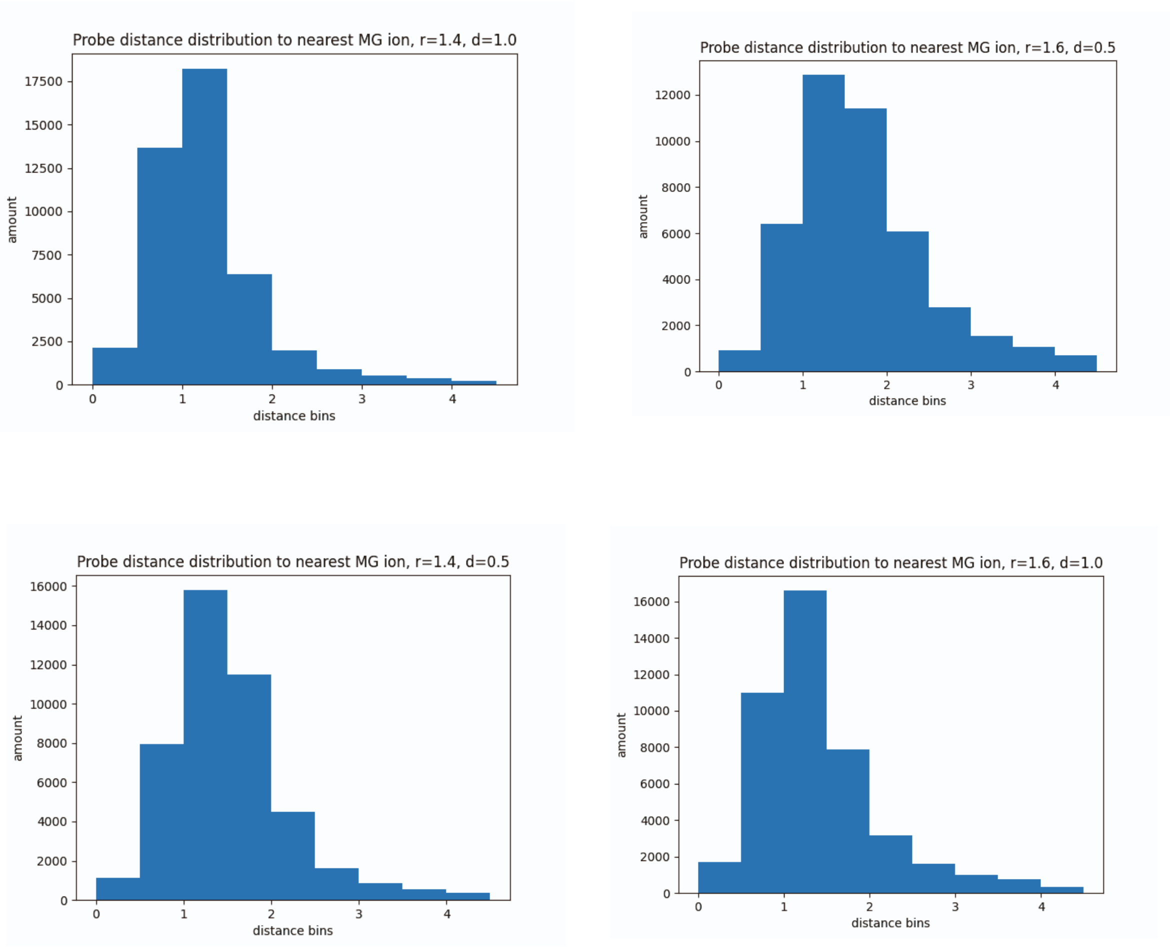


**Figure S6.** Probe distance from ground truth distribution according to different surface probe densities and probe radii of Connolly surface calculation method over all ions in our entire dataset.

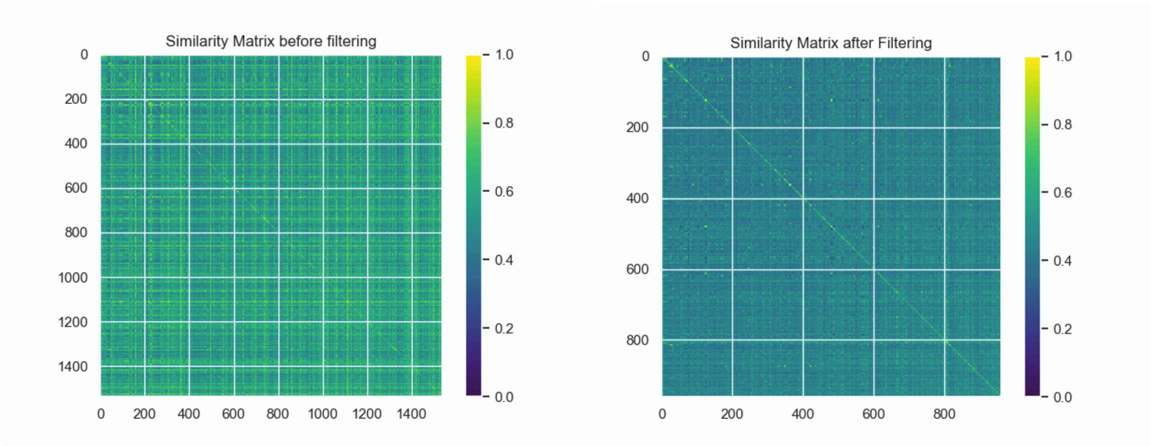


**Figure S7.** Pairwise sequence identity matrix between all RNA chains in the dataset (left) before filtering based on average sequence identity and (right) after filtering.

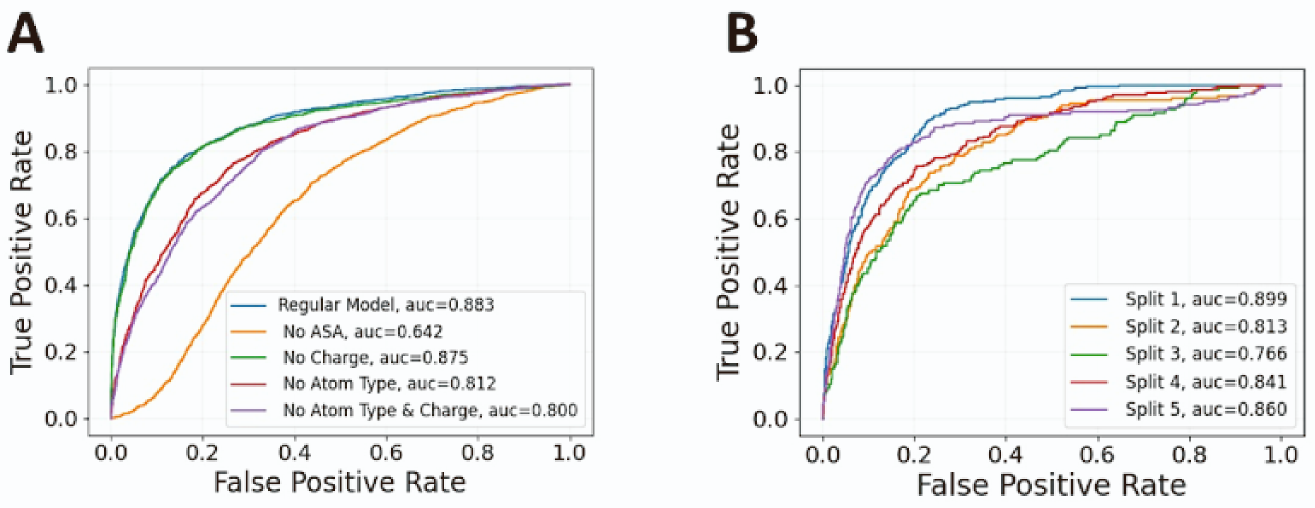


**Figure S8**. IonNet model metrics. A. ROC curves for our best model and ablation studies. B. Results of training using MetalIonRNA dataset.

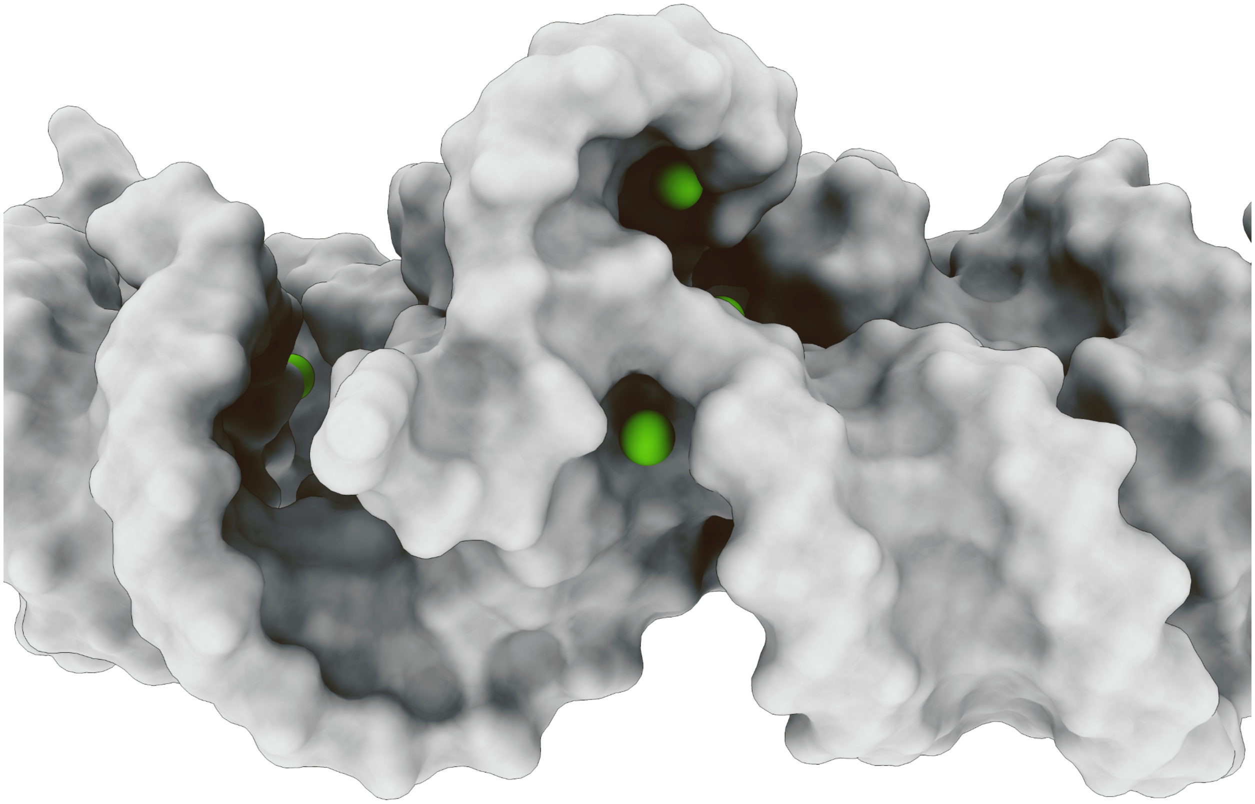

**Figure S9.** IonNet prediction sites in P4P6 structure. We find that IonNet prefers to place $Mg^{2+}$ ions in cavities on the RNA's surface.

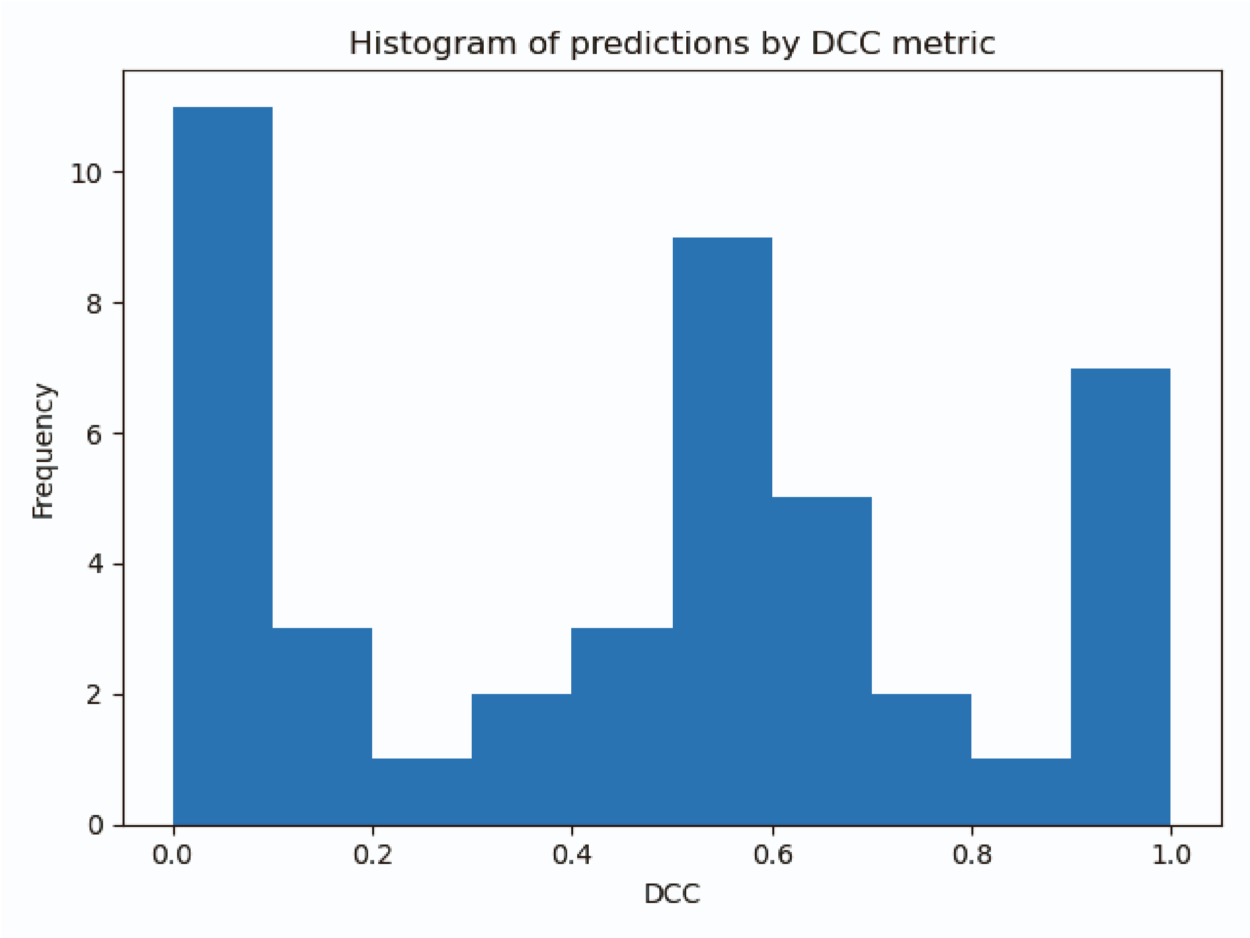


**Figure S10.** Distribution of DCC metric over the test set, with a weighted mean (by the number of $Mg^{2+}$ atoms per test case) of 0.46.

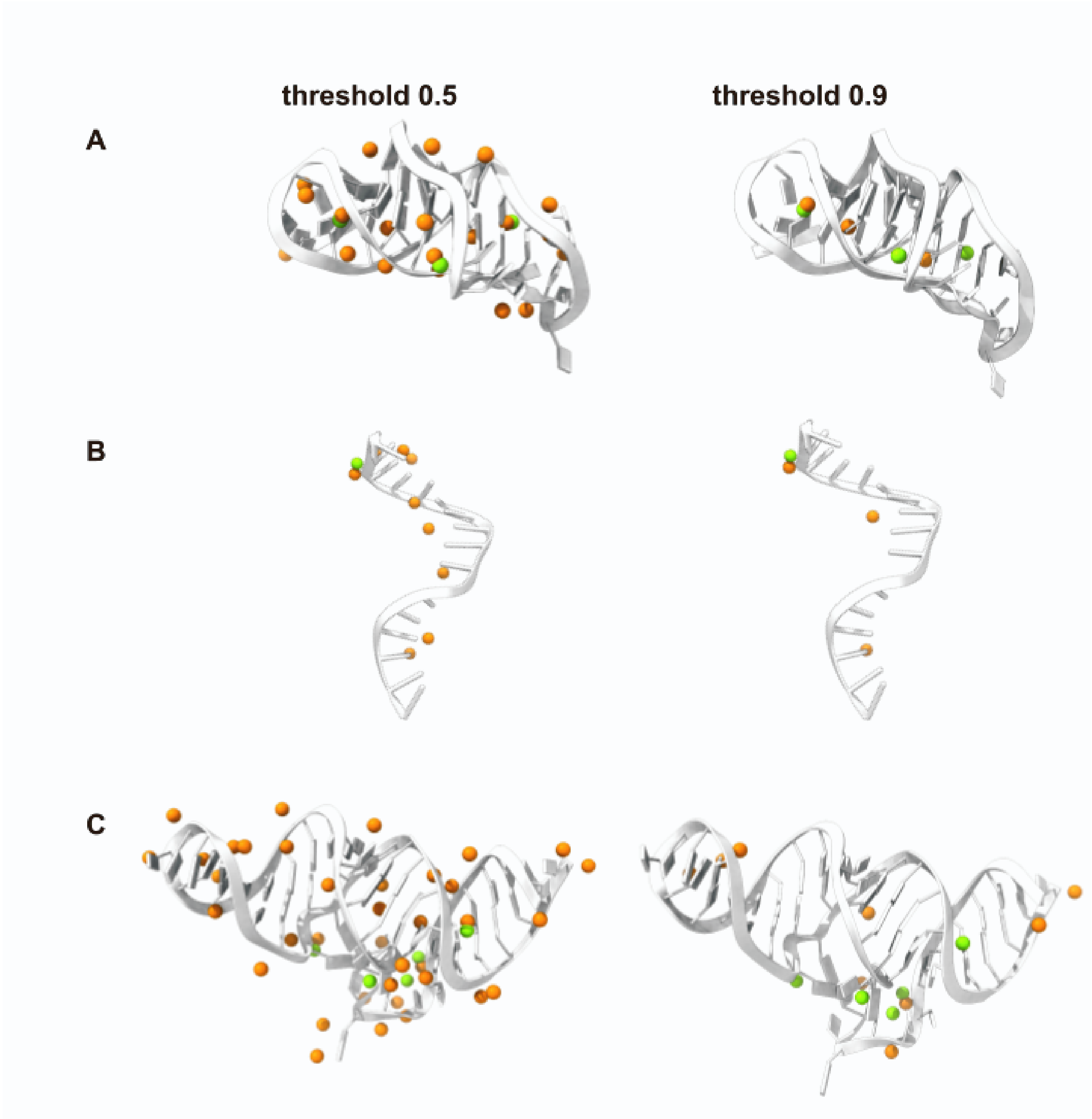


**Figure S11. A.** Guanidine III Riboswitch (PDB 5nz3, resolution 2.059Å), DCC score 0.6. IonNet predicted 18 $Mg^{2+}$ ion positions with a threshold of 0.5 after iterative clustering. Using a threshold of 0.9 resulted in 3 $Mg^{2+}$ ion positions without reducing the DCC score.

**B.** Guide RNA (PDB 6d8f, resolution 2.15Å), DCC score 1.0. IonNet predicted 9 $Mg^{2+}$ ion positions with a threshold of 0.5 after iterative clustering. Using a threshold of 0.9 resulted in 3 $Mg^{2+}$ ion positions without reducing the DCC score.

**C.** Structure of the ADP-binding domain of the NAD+ riboswitch (PDB 6tf2, resolution 2.55Å), DCC score 0.2. IonNet predicted 37 $Mg^{2+}$ ion positions with a threshold of 0.5 after iterative clustering. Using a threshold of 0.9 resulted in 9 $Mg^{2+}$ ion positions without reducing the DCC score.

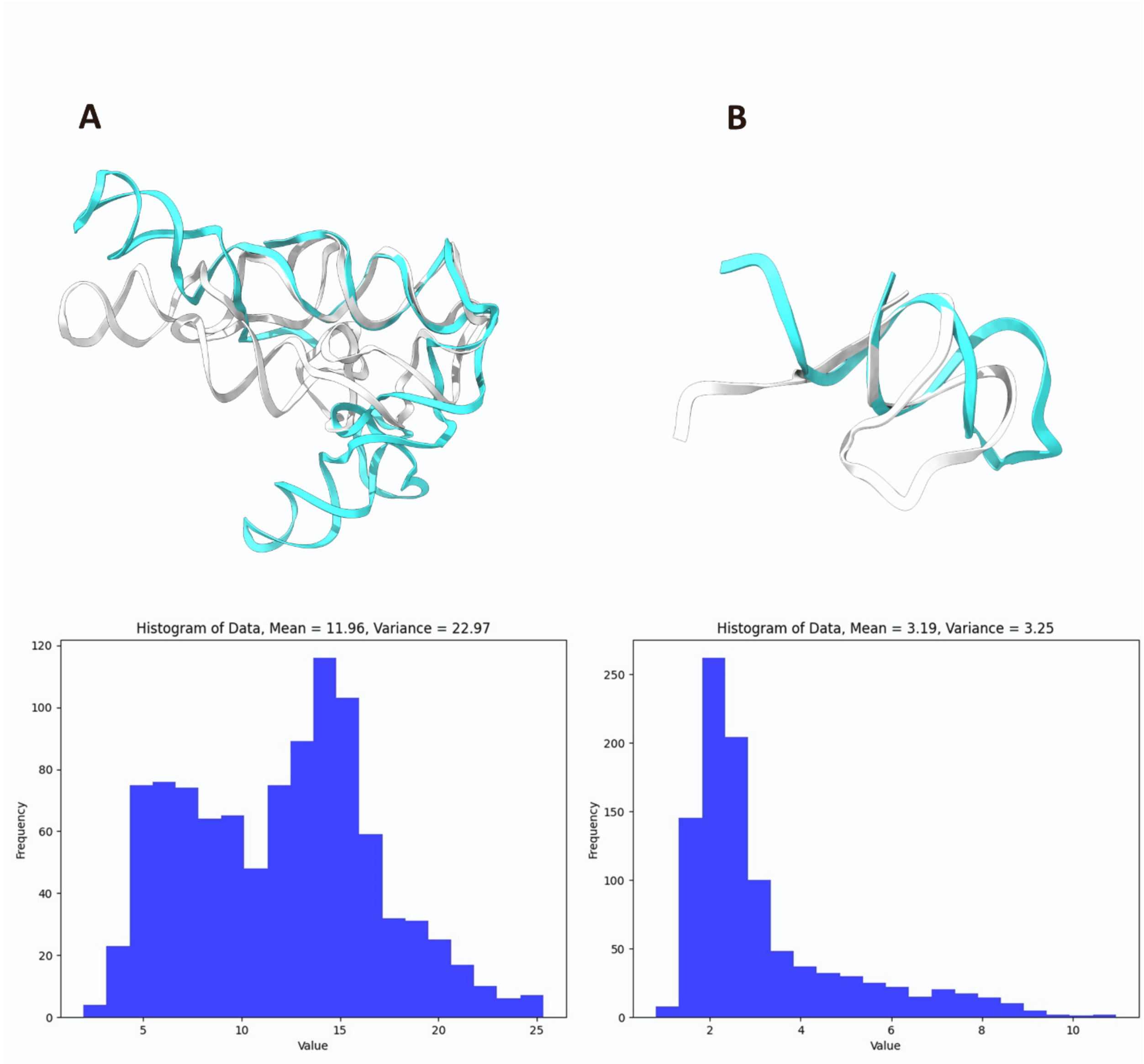


**Figure S12 Column A.** Sample #13 (P4P6) **Column B.** Sample #2 (JK11). Both columns have an image with initial structure (cyan) and the structure sampled by KGSRNA with highest RMSD structure when compared to the initial structure (white). Below the structure images is the distribution of RMSD between the original structure and all of the 1000 samples from KGSRNA.
